# A large‐scale targeted proteomics of plasma extracellular vesicles shows utility for prognosis prediction subtyping in colorectal cancer

**DOI:** 10.1002/cam4.5442

**Published:** 2022-11-16

**Authors:** Keiko Kasahara, Ryohei Narumi, Satoshi Nagayama, Keiko Masuda, Tsuyoshi Esaki, Kazutaka Obama, Takeshi Tomonaga, Yoshiharu Sakai, Yoshihiro Shimizu, Jun Adachi

**Affiliations:** ^1^ Department of Surgery Kyoto University Graduate School of Medicine Kyoto Japan; ^2^ Laboratory of Proteome Research National Institutes of Biomedical Innovation, Health and Nutrition Osaka Japan; ^3^ Laboratory of Proteomics for Drug Discovery Center for Drug Design Research, National Institute of Biomedical Innovation, Health and Nutrition Osaka Japan; ^4^ Laboratory of Clinical and Analytical Chemistry Collaborative Research Center for Health and Medicine, National Institute of Biomedical Innovation, Health and Nutrition Osaka Japan; ^5^ Department of Gastroenterological Surgery Gastroenterological Center, Cancer Institute Hospital, Japanese Foundation for Cancer Research Tokyo Japan; ^6^ Department of Surgery Uji‐Tokusyukai Medical Center Kyoto Japan; ^7^ Laboratory for Cell‐Free Protein Synthesis RIKEN Center for Biosystems Dynamics Research Osaka Japan; ^8^ The Center for Data Science Education and Research Shiga University Shiga Japan; ^9^ Japanese Red Cross Osaka Hospital Osaka Japan

**Keywords:** colorectal cancer, exosome, extracellular vesicle, mass spectrometry, targeted proteomics

## Abstract

**Purpose:**

The pathogenesis of cancers depends on the molecular background of each individual patient. Therefore, verifying as many biomarkers as possible and clarifying their relationships with each disease status would be very valuable. We performed a large‐scale targeted proteomics analysis of plasma extracellular vesicles (EVs) that may affect tumor progression and/or therapeutic resistance.

**Experimental design:**

Plasma EVs from 59 were collected patients with colorectal cancer (CRC) and 59 healthy controls (HC) in cohort 1, and 150 patients with CRC in cohort 2 for the large‐scale targeted proteomics analysis of 457 proteins as candidate CRC markers. The Mann–Whitney‐Wilcoxon test and random forest model were applied in cohort 1 to select promising markers. Consensus clustering was applied to classify patients with CRC in cohort 2. The Kaplan–Meier method and Cox regression analysis were performed to identify potential molecular factors contributing to the overall survival (OS) of patients.

**Results:**

In the analysis of cohort 1, 99 proteins were associated with CRC. The analysis of cohort 2 revealed two clusters showing significant differences in OS (*p* = 0.017). Twelve proteins, including alpha‐1‐acid glycoprotein 1 (ORM1), were suggested to be associated with the identified CRC subtypes, and ORM1 was shown to significantly contribute to OS, suggesting that ORM1 might be one of the factors closely related to the OS.

**Conclusions:**

The study identified two novel subtypes of CRC, which exhibit differences in OS, as well as important biomarker proteins that are closely related to the identified subtypes. Liquid biopsy assessment with targeted proteomics analysis was proposed to be crucial for predicting the CRC prognosis.

## Graphical abstract

Large‐scale selected reaction monitoring analysis of plasma extracellular vesicles from colorectal cancer patients targeting 457 proteins identified two subtypes of CRC patients. The subtypes were found to be different in overall survival. Putative protein biomarkers that are significantly associated with the subtypes were identified.

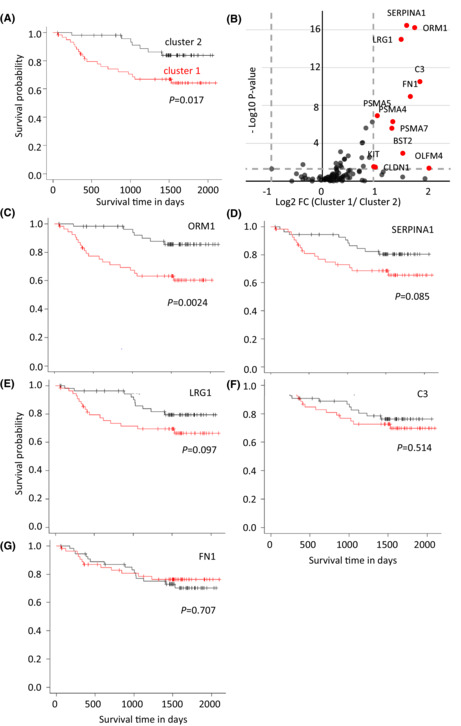

## 1 INTRODUCTION

Colorectal cancer (CRC) is the third most common cancer and the second leading cause of cancer‐related death worldwide.^1^ Currently, the TNM classification,^2^ which classifies malignant cancer based on clinicopathological findings, including an assessment of the primary tumor size (T), involvement of regional lymph nodes (N), and metastasis (M), is the gold standard for cancer staging and the subsequent determination of suitable therapeutic strategies. However, the field of diagnostics is shifting toward more detailed, molecular biological analyses. Recently, a CRC subtype classification system has been developed based on four consensus molecular subtypes, emphasizing the importance of prognostic predictions according to the individuals' molecular background for more personalized therapies.^2^, ^3^

Molecular analyses using tumor tissue samples generally necessitate invasive procedures, and repeat biopsies are often unbearable for patients. In contrast, blood collection is less invasive and is accepted as a surrogate for biopsy specimens, which is known as a liquid biopsy.^4^ A variety of targets for liquid biopsy, including circulating tumor cells, cell‐free DNA or RNA, and extracellular vesicles (EVs), have been proposed to date. Among them, EVs are suggested to be used for early cancer detection and prognosis.^5^ Every living cell is known to secrete EVs into body fluids. EVs have a biological function in cell–cell communication by carrying DNA, RNA, miRNAs and proteins, which affect tumor progression and/or treatment resistance.^6^ According to a recent report, a proteomics analysis of EVs is useful for cancer detection and cancer type determination, suggesting that EV protein profiles are ideal diagnostic tools.^5^

Mass spectrometry (MS)‐based proteomics studies provide unprecedented insights into proteomic profiles of biological samples without antibodies or other labeling techniques. Two major approaches are available for proteome profiling using MS: non‐targeted and targeted proteomics.^7^ Although non‐targeted proteomics mainly aims to identify as many proteins as possible and illustrate comprehensive protein landscapes in samples, targeted proteomics is used to quantify only target proteins to be measured. Due to these features, the former is generally used for the identification of biomarker candidates, and the latter is used for their verification in biomarker exploration studies.^5^, ^7^, ^8^

Due to the rapid progress of MS‐based biomarker discovery methods, there are often tens or hundreds of biomarker candidates. It is important to increase the number of proteins to be validated by targeted proteomics, which is one of the major challenges of this field and could lead to the discovery of more validated biomarkers. Targeted proteomics is commonly based on the technique called selected reaction monitoring (SRM) or multiple reaction monitoring (MRM).^9^ Data with a high signal‐to‐noise ratio are obtained by stabilizing only the ion traces with predefined *m/z* values utilizing triple quadrupole MS, enabling the highly sensitive detection and quantification of the target proteins with a wide dynamic range. For accurate quantification, particularly for the absolute quantification of proteins, stable isotope‐labeled (SIL) peptides are essential, as they have the same sequences as target peptides.^10^ However, the acquisition of SIL peptides is generally costly, and the preparation of various SIL peptides can be a bottleneck for large‐scale studies.

Accordingly, most typical biomarker studies have quantified less than 100 proteins for validation with SRM analysis.^5^, ^8^, ^11^, ^12^ Several studies have been reported as “large‐scale” SRM analyses. Ishizaki et al. developed an SRM assay of 135 biomarker candidate proteins for disease activity and organ involvement in anti‐neutrophil cytoplasmic antibody‐associated vasculitis.^13^ Kim et al. developed an assay of 133 prostate cancer biomarker candidate proteins using 232 peptides.^14^ You et al. developed an assay of 392 CRC‐related proteins using 641 peptides.^15^

Thus, in this study, we aimed to explore subtypes that explain the pathology or disease status of individual patients with CRC by analyzing plasma EVs, which were applied to the liquid biopsy assessment of the patients. Although various biomarker proteins in EVs have been reported to date,^5^, ^16^ we aimed to elucidate more validated molecular information by performing a large‐scale analysis with the highly sensitive SRM analysis. We also aimed to verify the significance of the obtained biomarkers by revealing their relationships with diagnostic information. A previously developed MS‐QBiC method^17^, ^18^ based on the multiplexed cell‐free synthesis of SIL peptides was applied, and over 1000 SIL peptides representing 457 proteins were prepared in a low‐cost and time‐saving manner. Two different cohorts were studied, and the analyses revealed the presence of protein biomarkers that could have a predictive and/or prognostic impact on CRC patients. Furthermore, machine learning‐based analysis suggested two subtypes of CRC that showed significant differences in overall survival (OS). Alpha‐1‐acid glycoprotein 1 (ORM1), an acute‐phase protein, was suggested to be strongly associated with the identified CRC subtypes with a poor prognosis. The findings obtained using a large‐scale targeted proteomics analysis of EVs may provide valuable insights into the prediction of the CRC prognosis.

## 2 MATERIALS AND METHODS

### 2.1 Sample collection

Plasma samples were collected from cohort 1 between April 2011 and December 2012 (patients with CRC) and October 2017 and July 2018 (healthy controls) at The Cancer Institute Ariake Hospital of Japanese Foundation of Cancer Research. Plasma samples were collected from cohort 2, comprising 150 patients with CRC of all disease stages, between September 2013 and December 2015 and were provided by Clinical Bio‐Resource Center, Kyoto University Hospital. See the SI Materials and Methods for details.

### 2.2 Sample preparation

EVs from plasma, peptide samples for MS analysis, and SIL peptides were prepared for this study. They were subjected to enzymatic digestion and prefractionation before MS analyses. See the SI Materials and Methods for details.

### 2.3 MS analyses

Two types of mass spectrometers, an Orbitrap mass spectrometer and a triple quadruple mass spectrometer, were used in this study. See the SI Materials and Methods for details.

### 2.4 Data analyses

Statistical analyses, random forest, consensus clustering, imputation of missing values, and correlation analyses of the obtained EV proteomic data were performed with R (http://cran.r‐project.org/). See the SI Materials and Methods for details.

## 3 RESULTS

### 3.1 Selection of the target proteins

**FIGURE 1:**
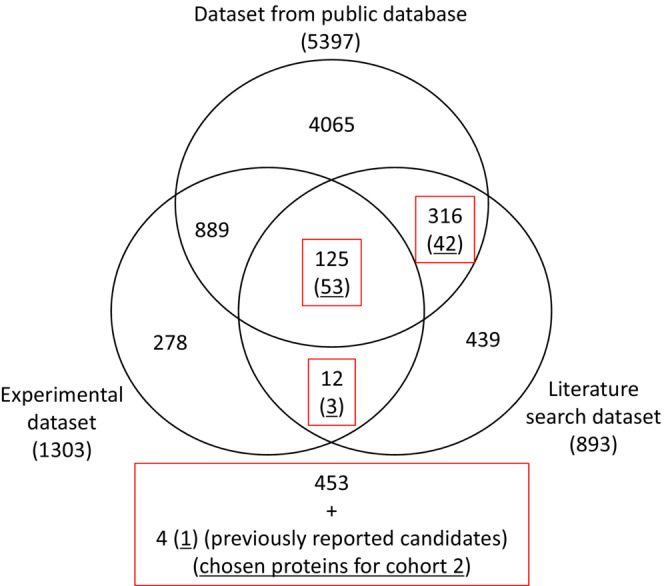
Selection of target proteins for the SRM analysis. A Venn diagram that includes the three datasets is shown. The selected proteins are boxed in red. The underlined number of proteins indicates the proteins selected for further analysis in cohort 2.

Two cohorts were analyzed according to our experimental design (Experimental design in the SI Results and discussions and Figure S1). The initial target proteins for the analysis of cohort 1 were selected from three datasets, including the literature search dataset (LS), experimental dataset (EP) and a dataset from a public database (PB) (Figure 1). LS contained 893 CRC biomarker candidate proteins that were reported in previous studies. The selection was performed for those published between 2003 and 2016, according to the same criteria as described previously.^11^ EP contained 1303 proteins, which were identified by non‐targeted proteomics analysis based on the mixtures of plasma samples in cohort 1. PB was additionally prepared to capture EV proteins not identified in the non‐targeted proteomics analysis. A total of 5397 proteins were collected from ExoCarta, a public database of exosomal proteins, RNA and lipids.^19^ All the proteins listed in the database as of June 2015 were collected after the removal of duplicates.

Further selection was performed principally based on the proteins listed in LS. LS proteins were prioritized because they have been reported to be functionally involved in CRC. Proteins identified in both LS and PB or LS and EP were selected as proteins to be analyzed (Figure 1). In addition, four proteins were added to the list, which were suggested to be putative CRC biomarker candidates in EVs,^11^ resulting in 457 proteins for the cohort 1 study.

For the SRM‐based targeted proteomics study, 1344 target peptides representing 457 proteins were designed (Data S2). Three peptides per protein were designed for all proteins. Peptide sequences registered in the public SRMAtlas database^20^ and those listed in EP were preferentially applied. Other peptides were designed according to the criteria used for peptide design in the MS‐QBiC method.^17^ All the designed SIL peptides were successfully synthesized with a reconstituted cell‐free protein synthesis system^21^ and used for SRM method development for the optimized monitoring of each peptide transition.

### 3.2 Identification of 99 CRC biomarker candidates through a large targeted analysis of cohort 1 EV samples

A total of 563 peptides representing 282 proteins were detected in the mixtures of plasma samples in cohort 1. Among them, 230 peptides representing 162 proteins were successfully quantified in the individual samples from cohort 1 (Datas S2, S3). The quantification limit of the endogenous signal was set to a < 1500 signal intensity to ensure quantification accuracy.

**FIGURE 2:**
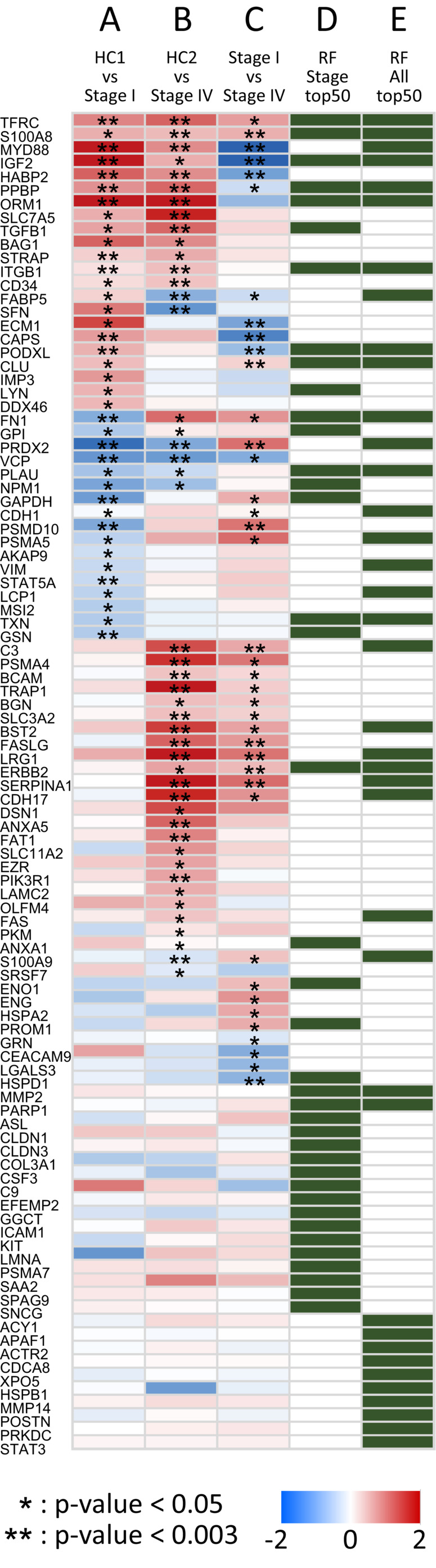
CRC biomarker candidate proteins identified in cohort 1. Comparisons of the relative amount of each protein in plasma EVs in the two groups, namely, between Stage I and HC1 (A), Stage IV and HC2 (B), and Stage I and Stage IV (C), are shown as a heat map. Colors ranging from blue to red represent log 2 (fold change), and asterisks indicate significance in the MWW tests (*p‐value <0.05. **p‐value <0.003). The top 50 proteins in the feature importance score in the RF analysis, derived from two datasets, Stages I and IV (D) and Stages I, IV, and HC1 and 2 (E), are shown in green panels.

Peptides that showed significant changes in the level quantified in plasma EVs between patients with CRC and healthy controls or patients with stage I and IV tumors were selected by statistical analyses, where the Mann–Whitney‐Wilcoxon (MWW) test was applied to the quantified values in each group. Comparisons were performed between Stage I and HC1 (A), Stage IV and HC2 (B), and Stage I and Stage IV (C). Peptides with p‐values less than 0.05 in the three comparative groups were listed as biomarker candidates (Figure 2).

Additionally, an unsupervised random forest (RF) analysis was performed on the quantified data from cohort 1 to evaluate the effect of the level of each peptide on each individual's status. Unsupervised RF was performed for two datasets, patient data (Stages I and IV) and all acquired data (Stages I, IV and HC1, 2). Multi‐dimensional scaling plots according to the similarities between samples in each dataset showed clustering of Stages I and IV (Figure S5a) and of patients and healthy controls (Figure S5b), respectively, suggesting the presence of factors that affect the differences between cancer stages or between patients and controls. The top 50 peptides in the feature importance score derived from two datasets, Stages I and IV (D) and Stages I and IV, and HC1 and 2 (E), were added to the candidates (Figure 2). Finally, 139 peptides representing 99 proteins were selected as biomarker candidates for the cohort 2 study (Data S2). The selected markers included proteins that may correlate with early diagnosis (A, E), distant metastasis (B, C, D), and disease progression (C, D).

### 3.3 Consensus clustering of proteomes in plasma EVs

To clarify the molecular backgrounds underlying disease status in each patient, that is, to investigate the relationships between each selected protein and clinicopathological observations, 99 selected biomarker candidates were further quantified in plasma EVs from 150 CRC patients in cohort 2 (Data S3). The patients were selected from another hospital over a certain period of time, regardless of the tumor stage; this external validation of candidate biomarkers aided in determining reproducibility and generalizability.

Exploratory cluster analysis was performed to visualize and interpret the cluster structures of the obtained data. To this end, consensus clustering (CC), a machine learning algorithm, was applied. CC has increased in popularity in cancer genomics, where new molecular subtypes of diseases have been identified to date.^22^, ^23^ This method involves multiple sub‐samplings from a set of data to form multiple clusters and determines the specified clusters. Therefore, it is a more robust approach that relies on multiple clustering for unsupervised samples even if noise, outliers or variations are included in the data.

We used the dataset of absolute levels of 99 proteins in 110 patients with CRC, after excluding data from 40 patients based on the eligibility criteria (see the Methods). Missing values that were not successfully quantified with the SRM analysis were replaced with half of the minimum protein level. The missing data mechanism in targeted proteomics is regarded as “missing not at random” values^24^ due to the limitations of MS detection sensitivity and specificity.

**FIGURE 3:**
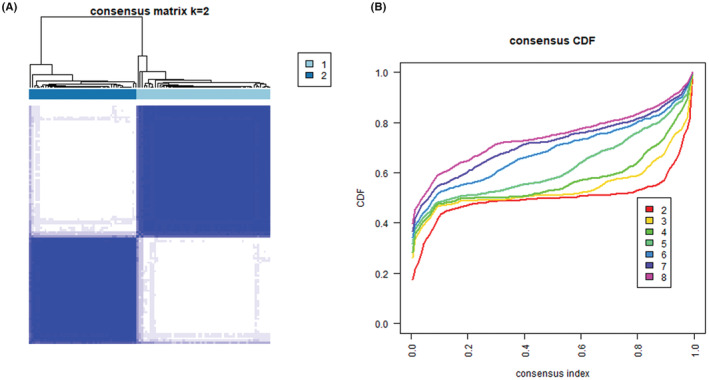
Consensus clustering of cohort 2 patients. The results of consensus clustering analysis are shown. (A) Consensus matrix plots when the maximum number of clusters (*K*) is set to two. (B) Empirical cumulative distribution function plots when the maximum number of clusters (*k*) is increased from two to eight.

A clear partitioning was observed in the consensus matrix (CM) plots at *k* = 2, where *k* represents the maximum number of clusters (Figure 3A). New small clusters appeared when *k* was increased from 3 to 5 (Figure S6a–c, g), and the two large clusters observed at *k* < 6 were divided into several clusters when *k* was further increased (Figure S6d–g). Empirical cumulative distribution function (CDF) plots showed a flat middle portion at *k* = 2 (Figure 3B). The proportion of ambiguous clustering (PAC), defined as the fraction of sample pairs with consensus index values falling in the specific intermediate sub‐interval (x_1_, x_2_), has been proposed to infer the optimal *k*,^25^ in which a low PAC value indicates a flat middle segment in CDF plots. As in the previous report, x_1_ and x_2_ were set to 0.1 and 0.9, respectively, and the PAC was calculated (Data S4), which showed the lowest value at *k* = 2, suggesting that the two major clusters observed at *k* = 2 are stable. Multiple methods of missing value imputation were further tested to confirm the clustering results. Reanalysis with several approaches showed almost the same clustering results, where two major stable clusters were observed (Figure S7–S9). From these results, we concluded that the obtained proteome data in cohort 2 contained two major clusters.

### 3.4 Clinical relevance of the two identified clusters

Characterization of the two major clusters identified in cohort 2 analysis was performed by investigating the relationship between the clustering and TNM stage of each individual. The results showed no apparent relationship with the clinicopathological classification, although more stage IV patients were included in cluster 1 (Figure S10). Patients of all stages were included in both clusters, suggesting that the proteomes in EVs are factors that are independent from clinical staging that can be used to classify CRC disease status.

**FIGURE 4:**
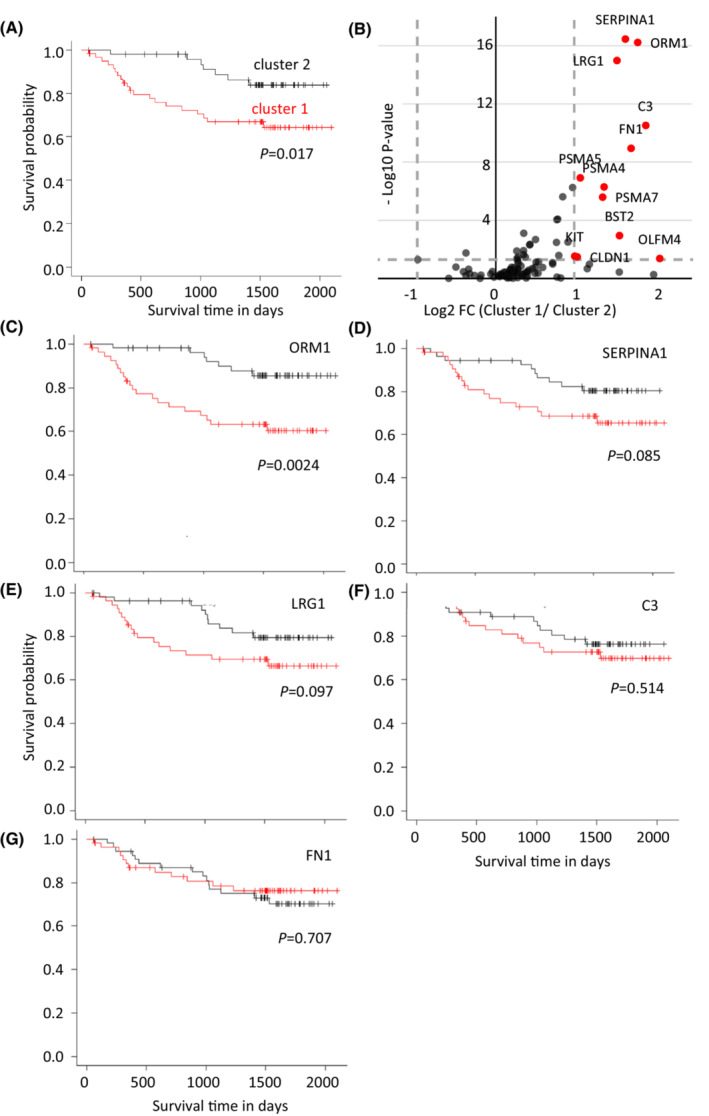
Characterization of the two clusters identified by consensus clustering. Differences between the two clusters were analyzed, with a focus on the overall survival (OS) of patients. (A) Kaplan–Meier OS curves of patients in two clusters. (B) Volcano plot for the comparison of protein levels between two clusters. The *X* axis is log 2 (fold change), and the *Y* axis is log 10 (*p*‐value). Twelve proteins in which the fold change was more than two and the *p*‐value was less than 0.05 are indicated by the red dots. (C–G) Kaplan–Meier OS curves of patients analyzed with a focus on specific proteins. The patients were divided into two groups based on whether (C) ORM1, (D) SERPINA1, (E) LRG1, (F) C3, or (G) FN1 was above or not the median level for each protein. The red and black curves indicate the groups with protein levels that were more or less than the median level, respectively.

The correlation between clustering and OS was analyzed by comparing Kaplan–Meier OS curves between clusters 1 and 2 to clarify this issue. Surprisingly, patients in cluster 1 experienced significantly shorter OS than those in cluster 2 (*p* = 0.017) (Figure 4A). Stage IV is the most important predictor of survival in CRC patients. The 5‐year relative survival rate for patients with stage IV CRC is approximately one‐sixth worse than that for patients with localized disease (14% vs. 90%).^26^ Because stage IV patients were more included in cluster 1 and it was possible that this difference affected the difference in OS between the two clusters, further analysis was performed with a focus on stage IV patients. Patients in cluster 1 experienced shorter OS than those in cluster 2 (Figure S11e, *p* = 0.245). Moreover, the rate of curative resection in cluster 1, which is strongly associated with better prognosis in Stage IV patients, was lower than that in cluster 2 (Data S5, Fisher's exact test, *p* = 0.14). Taken together, these results suggested that factors other than TNM staging may have been included in the clustering.

Proteins that showed significant differences in their levels in EVs between the two clusters were studied with a volcano plot analysis. Twelve upregulated proteins were found in cluster 1, in which fold changes were more than two and *p*‐values were less than 0.05, whereas no downregulated proteins were found (Figure 4B). Among the 12 proteins, 5 proteins, alpha‐1‐acid glycoprotein 1 (ORM1), alpha‐1‐antitrypsin (SERPINA1), leucine‐rich alpha‐2‐glycoprotein (LRG1), complement C3 (C3), and fibronectin (FN1), were successfully quantified in all samples from cohort 2 (Data S3); thus, they were further analyzed to reveal their correlations with OS.

All individuals in cohort 2 were divided into two groups based on whether each protein amount in the EVs was above the median value of the corresponding protein. Kaplan–Meier curves were applied for five proteins, and ORM1, SERPINA1, and LRG1 showed OS differences between the two groups (Figure 4C–G). In particular, ORM1 showed a statistically significant difference (*p* < 0.003), suggesting that ORM1 contributes greatly to OS.

Finally, Cox proportional hazards regression analysis, a multivariate analysis of OS, was performed on 110 patients in cohort 2, and variables including age, sex, TNM stage, and five proteins were examined. Two variables, TNM stage and ORM1, were significantly associated with shorter OS (*p* < 0.01) (Data S6). Because stage IV was the most important factor for OS, according to the statistical value in the multivariate analysis, ORM1 was further examined with only stage IV patients in cohort 2. The analysis again showed the importance of ORM1 in predicting shorter OS (Data S7), indicating that ORM1 in plasma EVs might be a factor independent of TNM staging that is closely related to shorter OS.

## 4 DISCUSSION

The present study revealed that a large‐scale targeted proteomics analysis identified several biomarker proteins in EVs that affect the prognosis of patients with CRC. We believe that the technical developments reported in the current study will further facilitate future exploratory studies in the field (See the SI Results and discussions). Furthermore, the machine learning‐based analyses identified two novel subtypes of CRC in patients that resulted in significant differences in OS. Because an apparent relationship between this classification and TNM staging was not observed, as patients with all stages of tumors were included in both subtypes (Figure S10), the developed subtyping method distinguish early‐stage patients who may transition to a more serious stage. Previous studies of biomarkers in tumor tissues have shown that the mutation status of genes known to be associated with CRC carcinogenesis (NRAS, KRAS, and BRAF) and defects in the DNA mismatch repair system, exert substantial effects on the treatment decision.^27^ However, obtaining such useful information requires invasive procedures that are difficult to perform at the first visit. Thus, the developed classification through a quantitative measurement of EV proteins in plasma might provide useful information to reinforce the gold standard TNM classification in determining the patient's future treatment plans. More detailed cell biological and biochemical analyses for elucidating the relationships between the identified biomarker proteins and the disease status are necessary to achieve this goal.

The analysis of cohort 1 provided a valuable list that may aid in investigating several aspects of CRC, including early diagnosis (A and E), distant metastasis (B, C, D), and disease progression (C, D), as shown in Figure 2. For example, TFRC, S100A8, MYD88, and IGF2 were suggested to be upregulated in EVs from patients with CRC, particularly in patients with stage IV tumors, with *p*‐values less than 0.01 in three MWW tests, and comparatively higher importance scores were obtained in either or both of the RF analyses. These proteins all have been previously reported to be upregulated in patients with CRC or other cancers,^28^, ^29^, ^30^, ^31^, ^32^ indicating the reliability of the present analyses. Considering that the cohort 1 analysis was performed with a highly sensitive SRM, which corresponds to a validated analysis in conventional integrative studies, the listed proteins are likely sufficient biomarkers to indicate disease status. Further analysis of the proteins showing significance in the statistical analyses, as well as those with large fold changes or high importance scores in the RF analysis, can provide more detailed information on the pathophysiological aspects of CRC from a molecular viewpoint.

The cohort 2 analysis showed that patients could be classified into two clusters, in which cluster 1 patients showed worse OS (Figures 3, 4). The difference in OS appears to be greater for stage IV patients than for other stage patients (Figure S11). the rate of achieving curative resection in patients with stage IV tumors was lower in cluster 1 (Data S5). These results indicate that this classification might be useful for predicting prolonged OS in stage IV patients by multidisciplinary approaches. Further studies with a larger cohort are needed to verify this aspect.

Twelve proteins were demonstrated to be upregulated in cluster 1, which had a shorter OS (Figure 4B). Among them, ORM1 was strongly suggested to be a significant protein to explain the shortened OS (Figure 4, Data S6, S7). ORM1 or alpha‐1‐acid glycoprotein 1 is a type of acute‐phase protein mainly biosynthesized and secreted by hepatocytes in response to an inflammatory systemic reaction.^33^ ORM1 expression and secretion have been reported to be increased in patients with several types of cancer, including hepatic carcinoma, gastric adenocarcinoma, epithelial ovarian cancer, lung cancer, and CRC.^34^, ^35^, ^36^, ^37^ ORM1 is one of the major serum glycoproteins and this protein may have been carried over in our isolated EV fractions. However, ORM1 is registered as an exosomal protein in ExoCarta^19^ because it was detected in urinary exosomes isolated via ultracetrifugation^38^; ORM1 has also been identified as one of the abundant proteins in EVs isolated via membrane affinity spin column from relapsed pediatric patients with Hodgkin lymphoma,^39^ and one of the tumor‐enriched EV proteins isolated via ultracentrifugation is present in patients with pancreatic adenocarcinoma.^5^ These previous studies strongly suggest that observed ORM1 upregulation in cluster 1 originated from EVs in the present study.

ORM1 mainly functions in acute‐phase reactions through its inflammatory and immunomodulatory properties,^33^ including the inhibition of neutrophil chemotaxis and superoxide production,^40^ lymphocyte proliferation,^41^ and platelet aggregation.^42^ It also antagonizes the capillary leakage caused by vascular permeability factors such as histamine and bradykinin.^43^ Thus, ORM1 in plasma EVs might modulate the immune system, which may be associated with the poor prognosis of patients with CRC.

**FIGURE 5:**
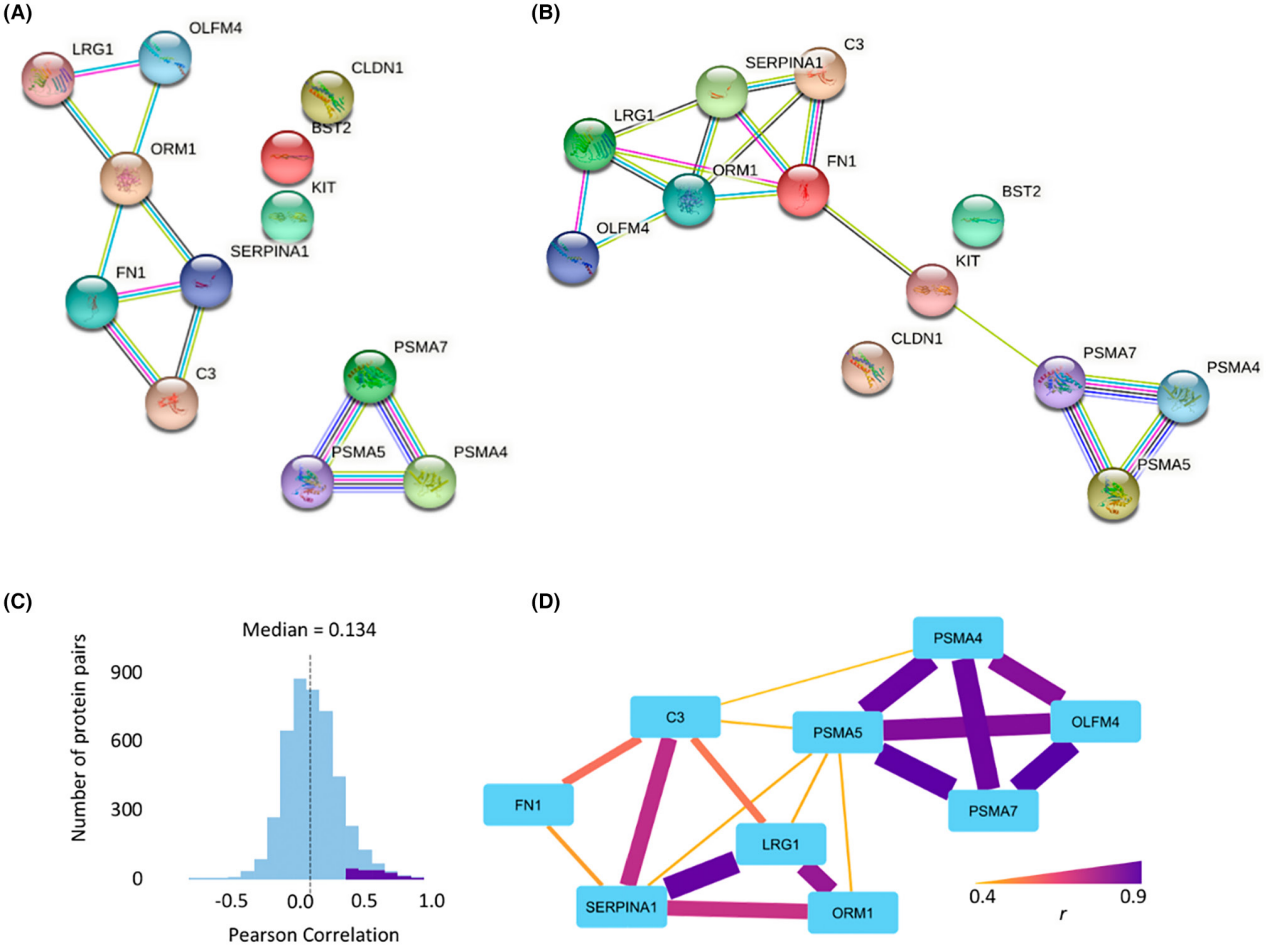
Predicted networks and correlation of upregulated proteins in cluster 1. Relationships among the 12 upregulated proteins in cluster 1 patients are shown. (A, B) Predicted PPI networks obtained using the STRING database^71^ with (A) high confidence and (B) medium confidence, respectively. (C) A histogram of the correlation coefficients between all pairs of 99 proteins analyzed in cohort 2. Purple pairs indicate the distribution of correlations with false discovery rate (FDR) < 1.0 × 10^−4^. (D) A correlation network of the upregulated proteins in cluster 1 according to the correlation analysis. Pairs with FDR < 1.0 × 10^−4^, including 12 upregulated proteins, were extracted and visualized with nodes and edges. The colors and thicknesses of the edges correspond to the correlation coefficients, indicating the strength of the correlation.

Interestingly, among the 11 proteins upregulated in cluster 1, except for ORM1, 8 proteins (BST2, FN1, LRG1, OLFM4, PSMA5, SERPINA1, C3, and KIT) were shown to share the exact same Gene Ontology (GO) biological process terms (acute‐phase response, inflammatory response, neutrophil degranulation, and platelet degranulation), according to the GO analysis using QuickGO (https://www.ebi.ac.uk/QuickGO/) (Data S9). Nine proteins also had immune‐related GO terms (Data S9), suggesting their associations with ORM1 in biological processes. Furthermore, the protein–protein interaction (PPI) network analysis performed with STRING (Search Tool for the Retrieval of Interacting Genes/Proteins) (https://string‐db.org/) indicated the presence of a cluster consisting of six proteins, including ORM1, with the highest confidence for the minimum required interaction score (Figure 5A). The cluster including ORM1 was further linked to the cluster including PSMA4, 5, and 7 via CLDN1 when the minimum required interaction score was set to medium confidence (Figure 5B).

Functional associations of these proteins were also suggested by the correlation analysis results of protein levels in the cohort 2 study. Correlation coefficients between all pairs in 99 proteins showed 181 (4.1%) significant positive correlations, where the false discovery rate was less than 1.0 × 10^−4^ (Figure 5C). Among them, 18 pairs were derived from 12 upregulated proteins (Data S10). The network structure visualized with nodes and edges (Figure 5D) showed good agreement with the GO and PPI network analyses. In particular, ORM1 has a strong correlation with LRG1 and SERPINA1 (*r* = 0.80 and 0.72, respectively), both of which share the same GO term (neutrophil degranulation) as ORM1 and have links in the PPI network analysis. Hence, both the informatics and experimental data strongly suggest that ORM1 collaboratively functions with cluster‐1 upregulated proteins.

EVs secreted from tumor tissues have been recently shown to play essential roles in remodeling the tumor immune microenvironment.^44^ Immunosuppressive signaling molecules in tumor‐derived EVs regulate the proliferation, maturation, and anti‐tumor capacity of targeted immune cells. Thus, immune‐related proteins in EVs might modulate the tumor immune microenvironment, potentially promoting CRC progression.

The results of the present study suggest that ORM1 is a strong biomarker that predicts the prognosis of CRC patients. Importantly, ORM1 and LRG1 are expressed at high levels in the urine of patients with adult‐onset Still's disease.^45^ EVs are known to be present in urine,^46^ and ORM1 in EVs may even be detected in the urine of patients with CRC. Thus, the evaluation of ORM1 levels in patient urine samples using high‐sensitivity SRM analysis has the potential to be a non‐invasive diagnostic method. Further studies are necessary to make SRM‐based quantitative proteomics applicable to clinical practice.

## 5 CONCLUSIONS

In this study, we described the relationships between diagnostic information, OS of patients with CRC, and the classified subtypes based on the quantitative molecular information. We consider that this linkage is important to achieve progress in determining the CRC diagnosis based on the identified biomarker proteins and tumor subtypes using our developed machine learning analysis and might predict prognosis of the patients; it might provide useful information to determine the patient's treatment plans. The mechanism underlying the associations between the identified proteins, including ORM1, with the prognosis of the patients remains unclear and we plan to elucidate this mechanism by performing cell biological and biochemical experiments in the future.

## AUTHOR CONTRIBUTIONS

Conceptualization: Keiko Kasahara, Ryohei Narumi, Satoshi Nagayama, Takeshi Tomonaga and Yoshihiro Shimizu; Experiment: Keiko Kasahara, Ryohei Narumi, and Keiko Masuda; Data analysis: Keiko Kasahara, Ryohei Narumi and Tsuyoshi Esaki; Manuscript preparation; Keiko Kasahara, Ryohei Narumi, Tsuyoshi Esaki and Yoshihiro Shimizu; Resource: Keiko Kasahara, Ryohei Narumi and Satoshi Nagayama; Supervision; Satoshi Nagayama, Kazutaka Obama, Yoshiharu Sakai, Yoshihiro Shimizu and Jun Adachi

## CONFLICT OF INTEREST

The named authors have no conflict of interest, financial or otherwise.

## ETHICS STATEMENT

Approval of the research protocol by an Institutional Reviewer Board. Registry and the Registration No. of the studies. The study was approved by the Ethics Committee of The Cancer Institute Ariake Hospital of Japanese Foundation of Cancer Research (2010–1058) and Kyoto University Hospital (G1077).

## INFORMED CONSENT

Informed consent was obtained from the patients and healthy controls at The Cancer Institute Ariake Hospital of Japanese Foundation of Cancer Research in the form of opt‐out on the web‐site at Kyoto University Hospital.

## ANIMAL STUDIES

Not available.

## Supporting information


Data S1

Data S2

Data S3

Data S4

Data S5

Data S6

Data S7

Data S8

Data S9

Data S10

Figure S1

Figure S2

Figure S3

Figure S4

Figure S5

Figure S6

Figure S7

Figure S8

Figure S9

Figure S10

Figure S11

Figure S11

## Data Availability

Raw data and result files that support the findings of this study have been deposited in jPOST (https://jpostdb.org) with the accession codes JPST000752/PXD018228.
