# Supplementary material for: A large‐scale targeted proteomics of plasma extracellular vesicles shows utility for prognosis prediction subtyping in colorectal cancer
Source: Cancer Med. 2022 Nov 16;12(6):7616–26. doi: 10.1002/cam4.5442 (PMC10067095; doi:10.1002/cam4.5442)
Supplement: Supplementary file 11 — Figure S1 [file CAM4-12-7616-s020.pptx]

## Slide 1
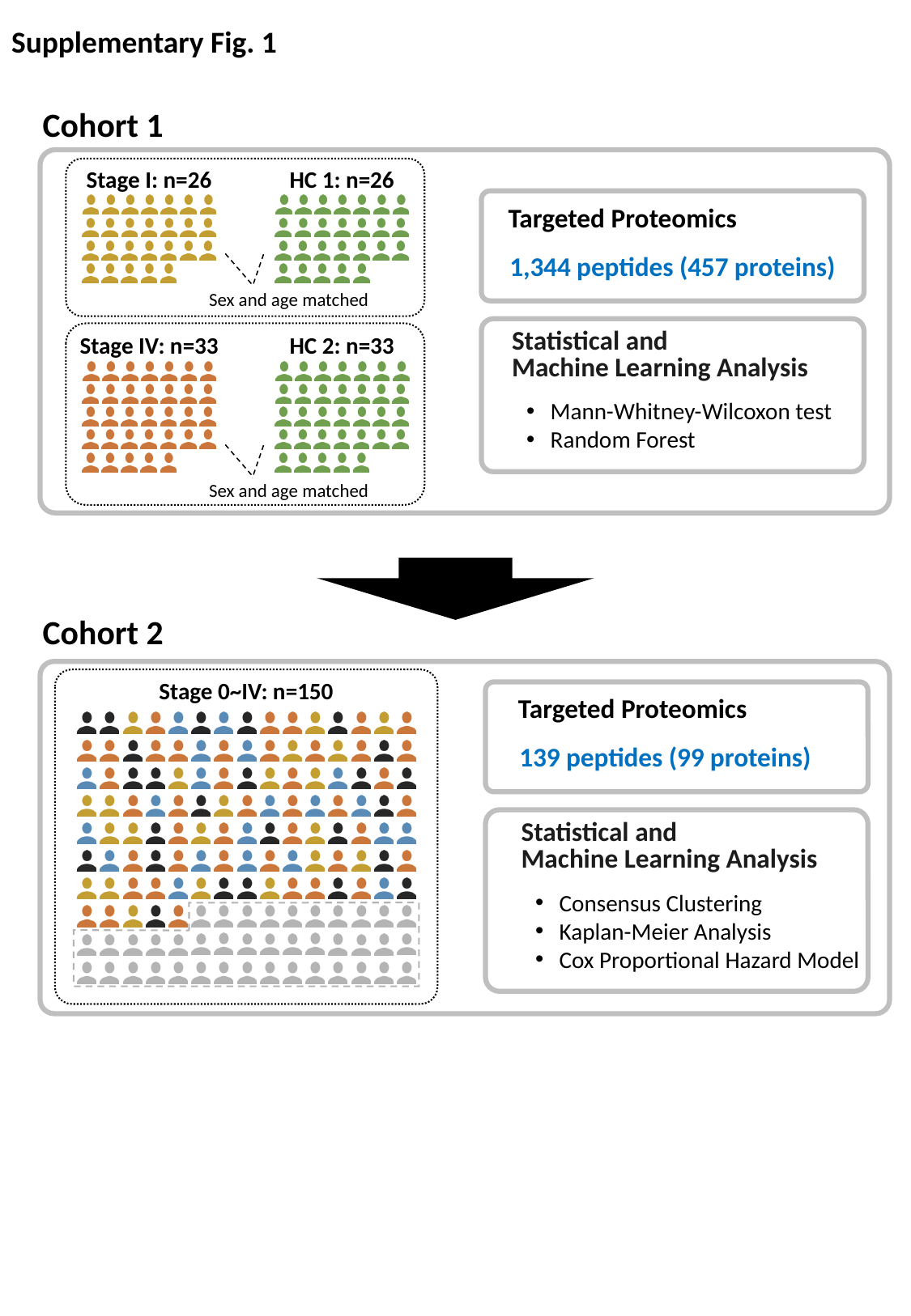

Supplementary Fig. 1
Cohort 1
Stage I: n=26
HC 1: n=26
Targeted Proteomics
1,344 peptides (457 proteins)
Sex and age matched
Statistical and
Machine Learning Analysis
Mann-Whitney-Wilcoxon test
Random Forest
Stage IV: n=33
HC 2: n=33
Sex and age matched
Cohort 2
Stage 0~IV: n=150
Targeted Proteomics
139 peptides (99 proteins)
Statistical and
Machine Learning Analysis
Consensus Clustering
Kaplan-Meier Analysis
Cox Proportional Hazard Model
