# Supplementary material for: A large‐scale targeted proteomics of plasma extracellular vesicles shows utility for prognosis prediction subtyping in colorectal cancer
Source: Cancer Med. 2022 Nov 16;12(6):7616–26. doi: 10.1002/cam4.5442 (PMC10067095; doi:10.1002/cam4.5442)
Supplement: Supplementary file 12 — Figure S2 [file CAM4-12-7616-s006.pptx]

## Slide 1
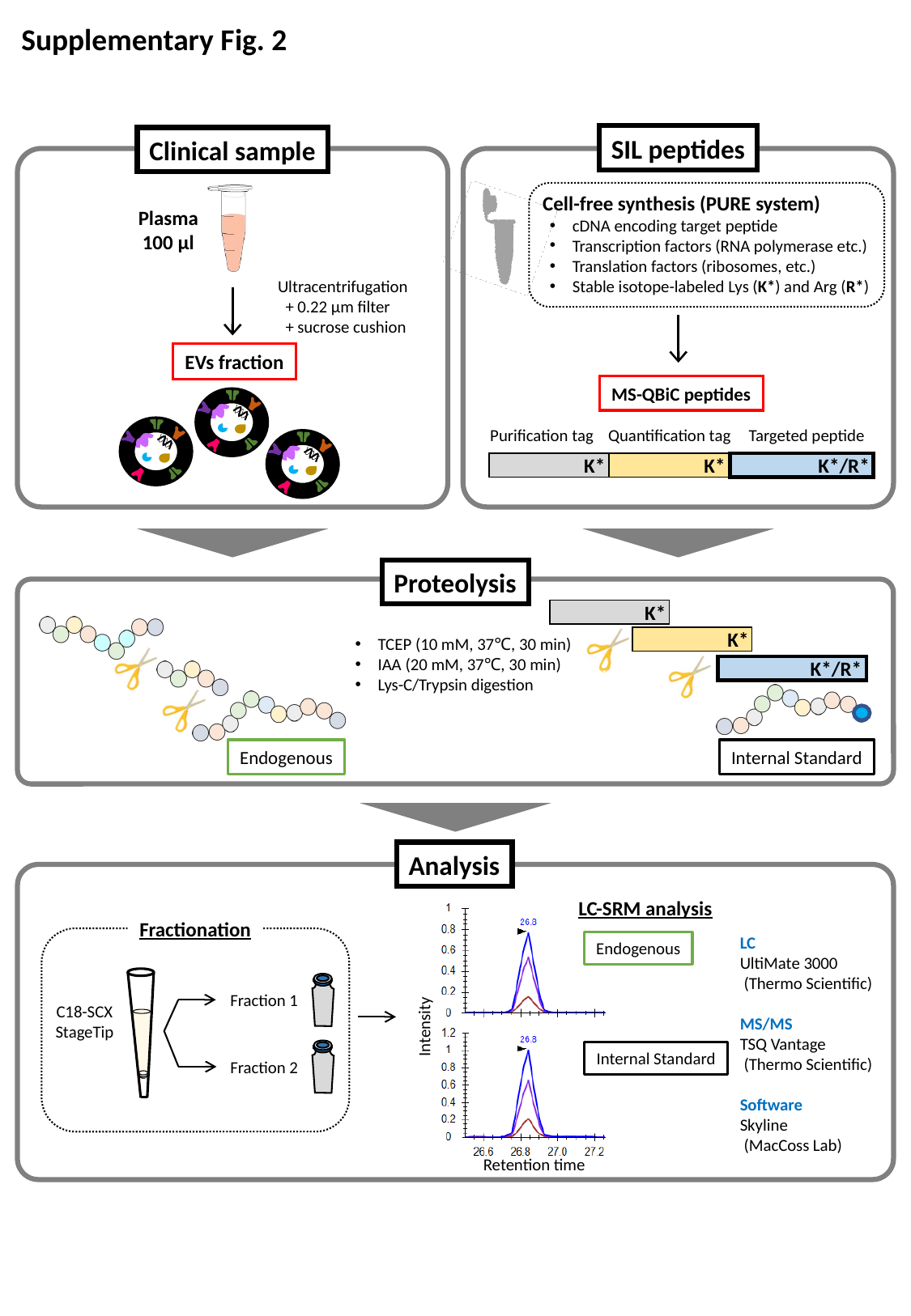

Supplementary Fig. 2
SIL peptides
Clinical sample
Cell-free synthesis (PURE system)
Plasma
100 µl
cDNA encoding target peptide
Transcription factors (RNA polymerase etc.)
Translation factors (ribosomes, etc.)
Stable isotope-labeled Lys (K*) and Arg (R*)
Ultracentrifugation
 + 0.22 µm filter
 + sucrose cushion
EVs fraction
MS-QBiC peptides
Purification tag
Quantification tag
Targeted peptide
K*
K*
K*/R*
Proteolysis
K*
K*
TCEP (10 mM, 37℃, 30 min)
IAA (20 mM, 37℃, 30 min)
Lys-C/Trypsin digestion
K*/R*
Endogenous
Internal Standard
Analysis
LC-SRM analysis
Fractionation
LC
UltiMate 3000
 (Thermo Scientific)
MS/MS
TSQ Vantage
 (Thermo Scientific)
Software
Skyline
 (MacCoss Lab)
Endogenous
Fraction 1
C18-SCX
StageTip
Intensity
Internal Standard
Fraction 2
Retention time
