# Supplementary material for: A large‐scale targeted proteomics of plasma extracellular vesicles shows utility for prognosis prediction subtyping in colorectal cancer
Source: Cancer Med. 2022 Nov 16;12(6):7616–26. doi: 10.1002/cam4.5442 (PMC10067095; doi:10.1002/cam4.5442)
Supplement: Supplementary file 13 — Figure S3 [file CAM4-12-7616-s005.pptx]

## Slide 1
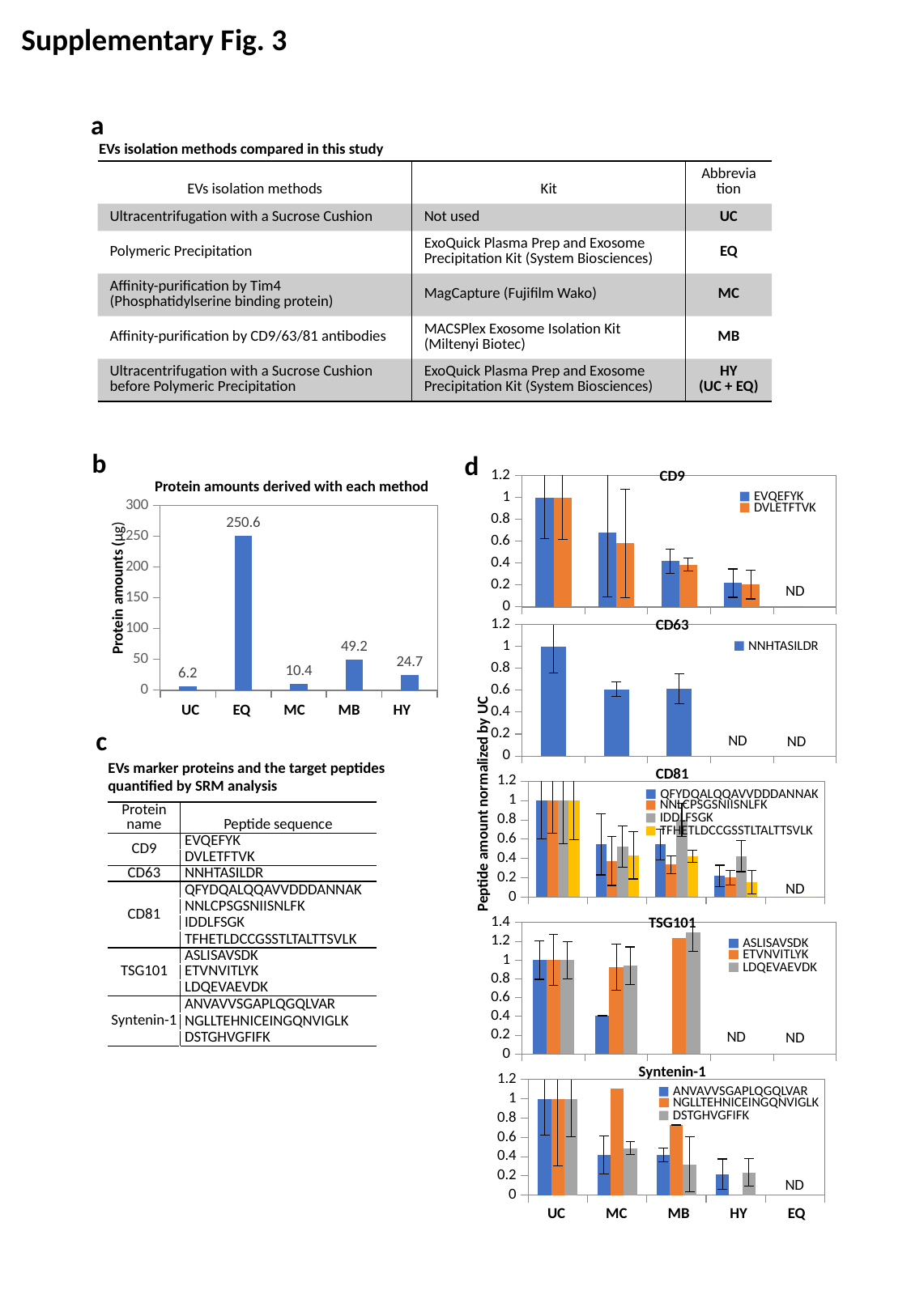

Supplementary Fig. 3
a
EVs isolation methods compared in this study
| EVs isolation methods | Kit | Abbreviation |
| --- | --- | --- |
| Ultracentrifugation with a Sucrose Cushion | Not used | UC |
| Polymeric Precipitation | ExoQuick Plasma Prep and Exosome Precipitation Kit (System Biosciences) | EQ |
| Affinity-purification by Tim4 (Phosphatidylserine binding protein) | MagCapture (Fujifilm Wako) | MC |
| Affinity-purification by CD9/63/81 antibodies | MACSPlex Exosome Isolation Kit (Miltenyi Biotec) | MB |
| Ultracentrifugation with a Sucrose Cushion before Polymeric Precipitation | ExoQuick Plasma Prep and Exosome Precipitation Kit (System Biosciences) | HY (UC + EQ) |
b
d
CD9
### Chart
| Category | EVQEFYK | DVLETFTVK |
|---|---|---|
| UC | 1.0 | 1.0 |
| MC | 0.6769094028162177 | 0.5803960485620318 |
| MB | 0.41651562113730295 | 0.38475926038536995 |
| HY | 0.21730685000714894 | 0.20314563605733577 |
| EQ | 0.0 | 0.0 |Protein amounts derived with each method
EVQEFYK
DVLETFTVK
### Chart
| Category | |
|---|---|
| UC | 6.2 |
| EQ | 250.6 |
| MC | 10.4 |
| MB | 49.2 |
| HY | 24.7 |UC
EQ
MC
MB
HY
Protein amounts (µg)
ND
ND
ND
ND
ND
ND
ND
UC
MC
MB
HY
EQ
CD63
### Chart
| Category | NNHTASILDR |
|---|---|
| UC | 1.0 |
| MC | 0.6082782886236259 |
| MB | 0.614900710662841 |
| HY | None |
| EQ | None |NNHTASILDR
c
EVs marker proteins and the target peptides quantified by SRM analysis
CD81
### Chart
| Category | QFYDQALQQAVVDDDANNAK | NNLCPSGSNIISNLFK | IDDLFSGK | TFHETLDCCGSSTLTALTTSVLK |
|---|---|---|---|---|
| UC | 1.0 | 1.0 | 1.0 | 1.0 |
| MC | 0.5451597487116551 | 0.37451214574380076 | 0.5265025173179991 | 0.4348575481046027 |
| MB | 0.547959855587875 | 0.33708500464107277 | 0.8018893959934124 | 0.4245703518751975 |
| HY | 0.21793211629579742 | 0.20169255120274232 | 0.42519280837777707 | 0.15600240303571283 |
| EQ | 0.0 | 0.0 | 0.0 | 0.0 |QFYDQALQQAVVDDDANNAK
NNLCPSGSNIISNLFK
IDDLFSGK
TFHETLDCCGSSTLTALTTSVLK
| | |
| --- | --- |
| Protein name | Peptide sequence |
| CD9 | EVQEFYK |
| | DVLETFTVK |
| CD63 | NNHTASILDR |
| CD81 | QFYDQALQQAVVDDDANNAK |
| | NNLCPSGSNIISNLFK |
| | IDDLFSGK |
| | TFHETLDCCGSSTLTALTTSVLK |
| TSG101 | ASLISAVSDK |
| | ETVNVITLYK |
| | LDQEVAEVDK |
| Syntenin-1 | ANVAVVSGAPLQGQLVAR |
| | NGLLTEHNICEINGQNVIGLK |
| | DSTGHVGFIFK |
| | |
Peptide amount normalized by UC
TSG101
### Chart
| Category | ASLISAVSDK | ETVNVITLYK | LDQEVAEVDK |
|---|---|---|---|
| UC | 1.0 | 1.0 | 1.0 |
| MC | 0.4085215534389376 | 0.9230192322723587 | 0.9402709886405537 |
| MB | None | 1.2320071070223617 | 1.2963012016248558 |
| HY | None | None | None |
| EQ | None | None | None |ASLISAVSDK
ETVNVITLYK
LDQEVAEVDK
Syntenin-1
### Chart
| Category | ANVAVVSGAPLQGQLVAR | NGLLTEHNICEINGQNVIGLK | DSTGHVGFIFK |
|---|---|---|---|
| UC | 1.0 | 1.0 | 1.0 |
| MC | 0.4149633167765394 | 1.1049155664674652 | 0.4877614129685731 |
| MB | 0.4185567328199376 | 0.7306703331094011 | 0.31947605172461907 |
| HY | 0.21631694040038502 | None | 0.23613724484757695 |
| EQ | None | None | None |ANVAVVSGAPLQGQLVAR
NGLLTEHNICEINGQNVIGLK
DSTGHVGFIFK
