# Supplementary material for: A large‐scale targeted proteomics of plasma extracellular vesicles shows utility for prognosis prediction subtyping in colorectal cancer
Source: Cancer Med. 2022 Nov 16;12(6):7616–26. doi: 10.1002/cam4.5442 (PMC10067095; doi:10.1002/cam4.5442)
Supplement: Supplementary file 14 — Figure S4 [file CAM4-12-7616-s015.pptx]

## Slide 1
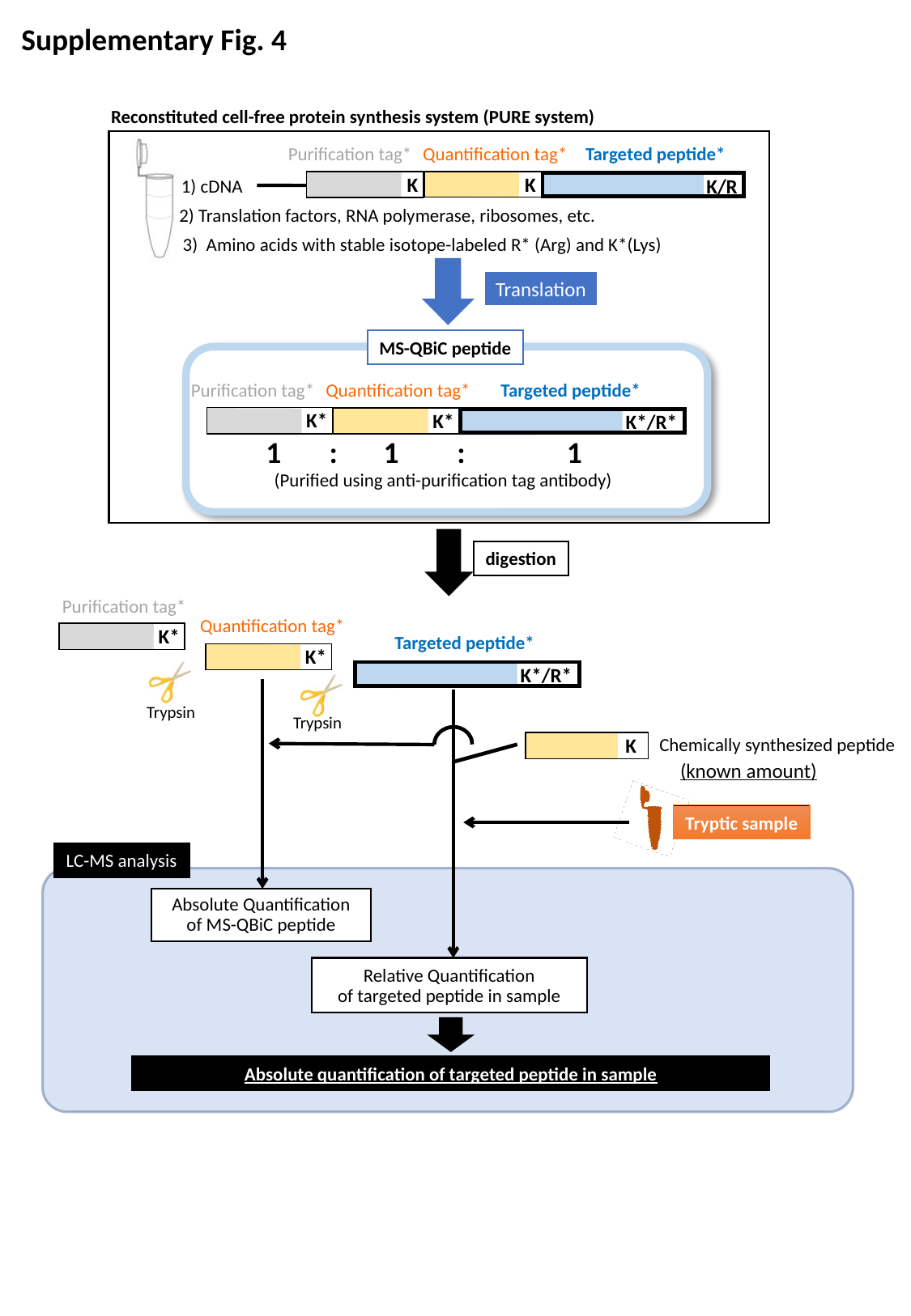

Supplementary Fig. 4
Reconstituted cell-free protein synthesis system (PURE system)
Purification tag*
Quantification tag*
Targeted peptide*
K
K
K/R
1) cDNA
2) Translation factors, RNA polymerase, ribosomes, etc.
3) Amino acids with stable isotope-labeled R* (Arg) and K*(Lys)
Translation
MS-QBiC peptide
Purification tag*
Quantification tag*
Targeted peptide*
K*
K*
K*/R*
1
:
1
:
1
(Purified using anti-purification tag antibody)
digestion
Purification tag*
Quantification tag*
K*
Targeted peptide*
K*
K*/R*
Trypsin
Trypsin
K
Chemically synthesized peptide
(known amount)
Tryptic sample
LC-MS analysis
Absolute Quantification
of MS-QBiC peptide
Relative Quantification
of targeted peptide in sample
Absolute quantification of targeted peptide in sample
