# Supplementary figures and images for: A large‐scale targeted proteomics of plasma extracellular vesicles shows utility for prognosis prediction subtyping in colorectal cancer
Source: Cancer Med. 2022 Nov 16;12(6):7616–26. doi: 10.1002/cam4.5442 (PMC10067095; doi:10.1002/cam4.5442)

## Slide 1
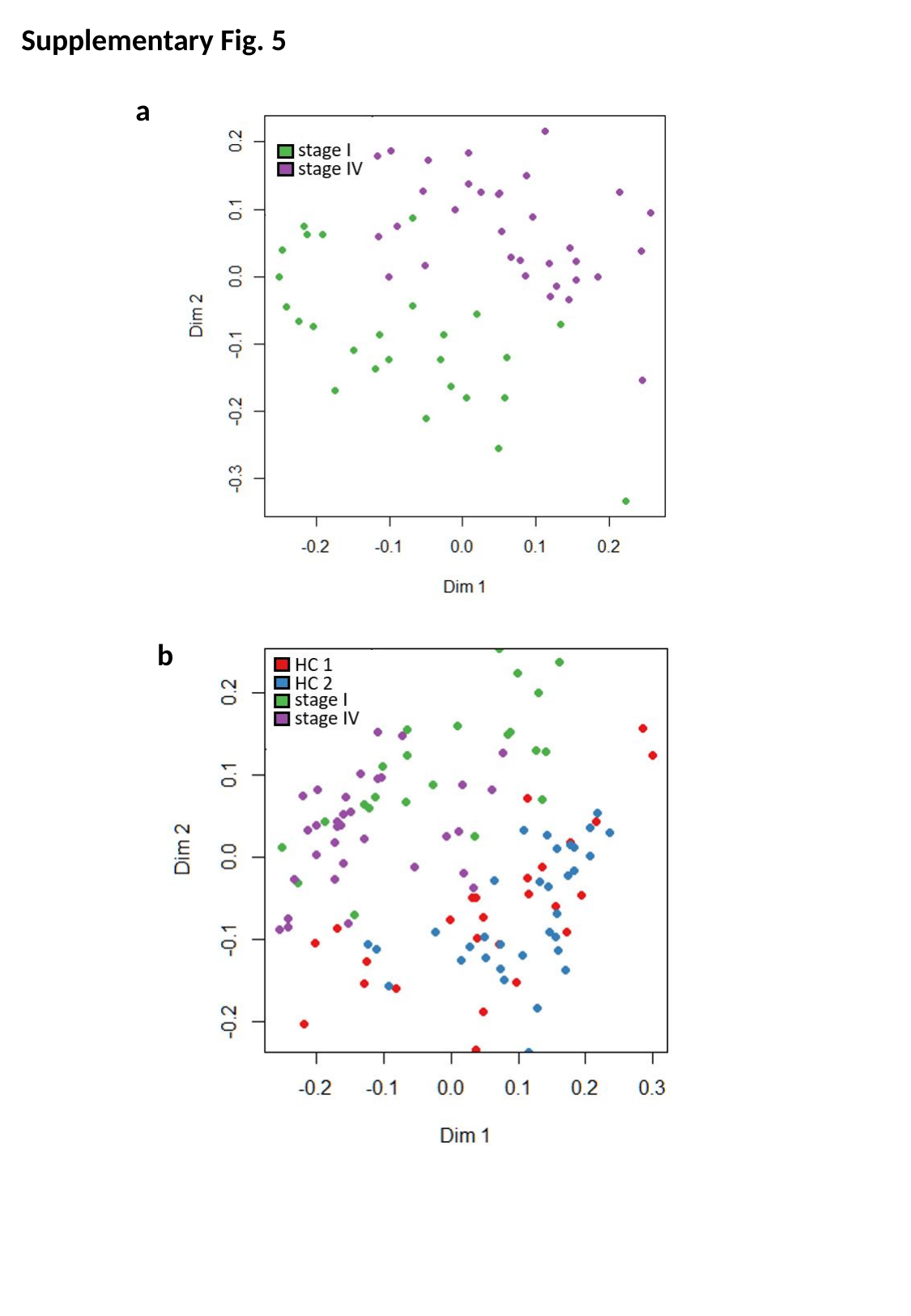

Supplementary Fig. 5
a
b

Supplement: Supplementary file 15 — Figure S5 [file CAM4-12-7616-s012.pptx]

## Slide 1
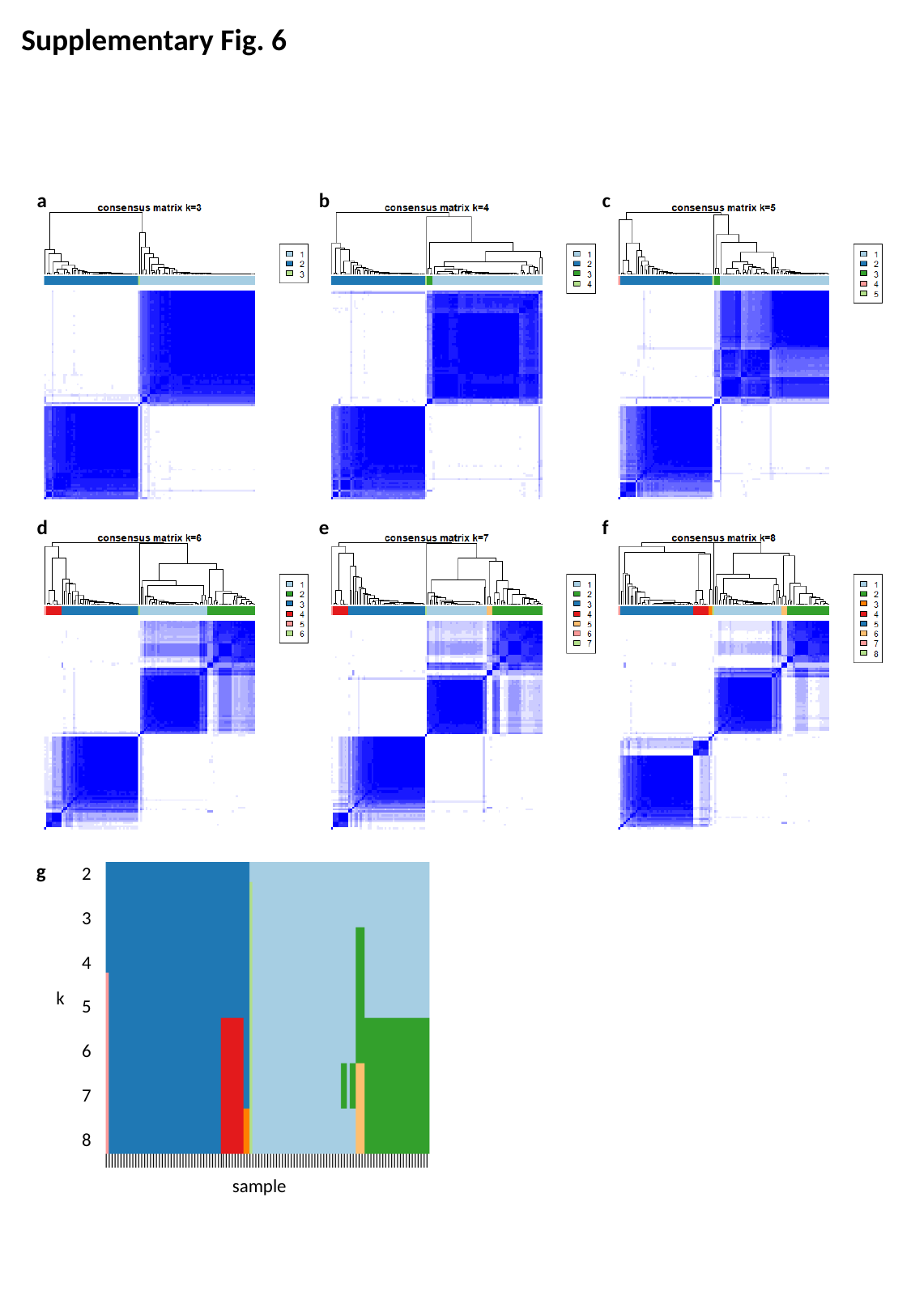

Supplementary Fig. 6
a
b
c
d
e
f
g
2
3
4
5
6
7
8
k
sample

Supplement: Supplementary file 16 — Figure S6 [file CAM4-12-7616-s010.pptx]

## Slide 1
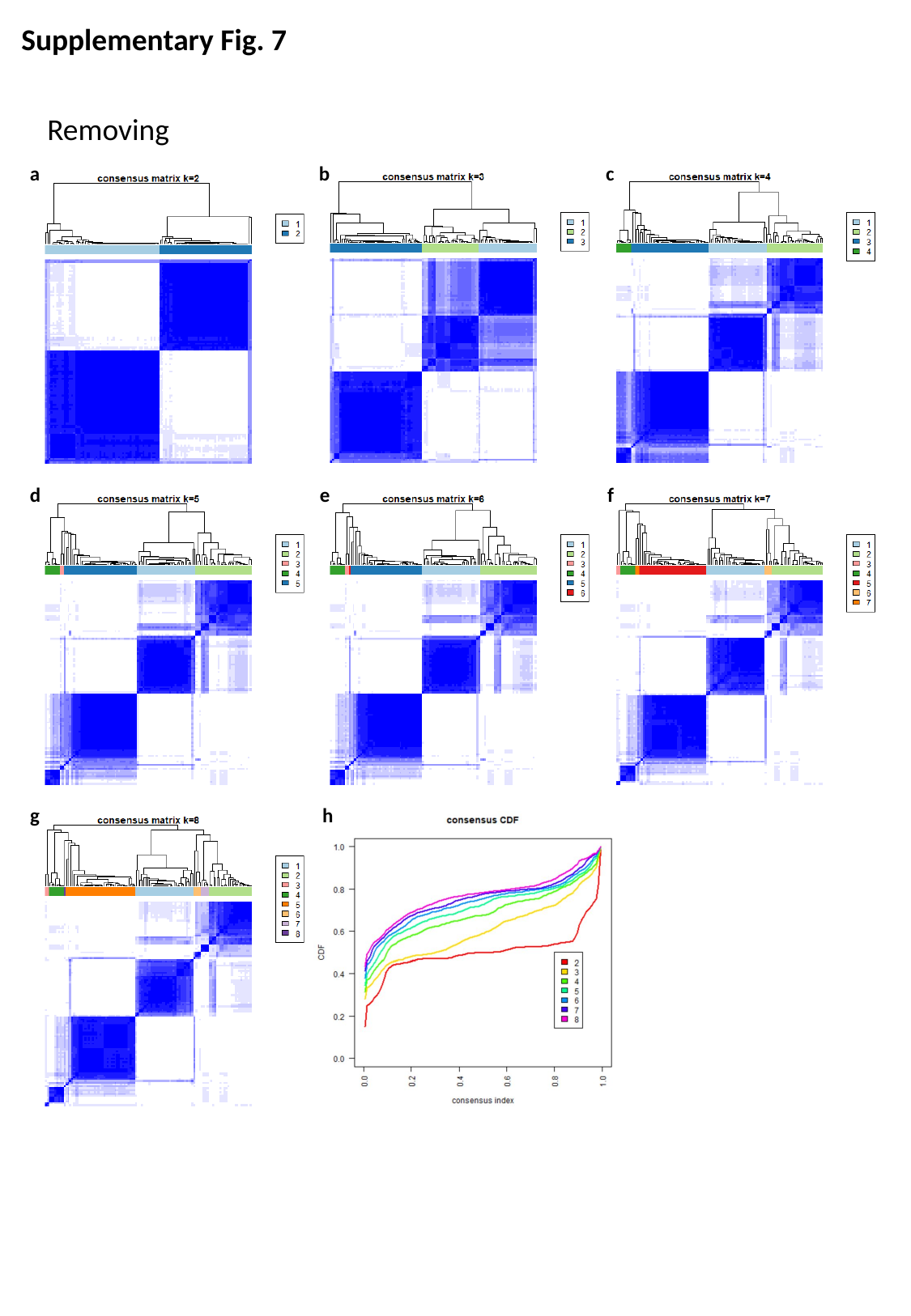

Supplementary Fig. 7
Removing
a
b
c
d
e
f
g
h

Supplement: Supplementary file 17 — Figure S7 [file CAM4-12-7616-s016.pptx]

## Slide 1
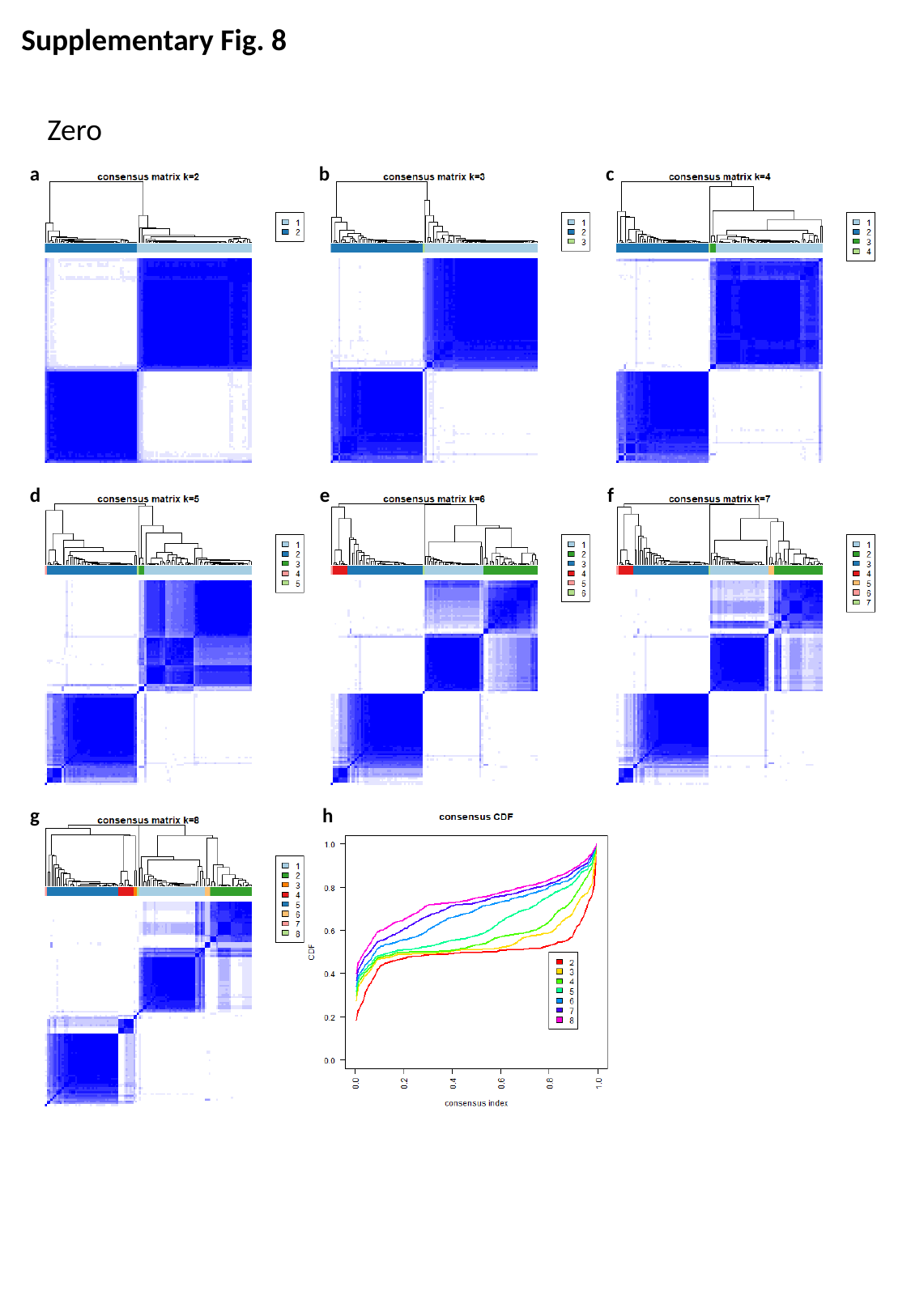

Supplementary Fig. 8
Zero
a
b
c
d
e
f
g
h

Supplement: Supplementary file 18 — Figure S8 [file CAM4-12-7616-s004.pptx]

## Slide 1
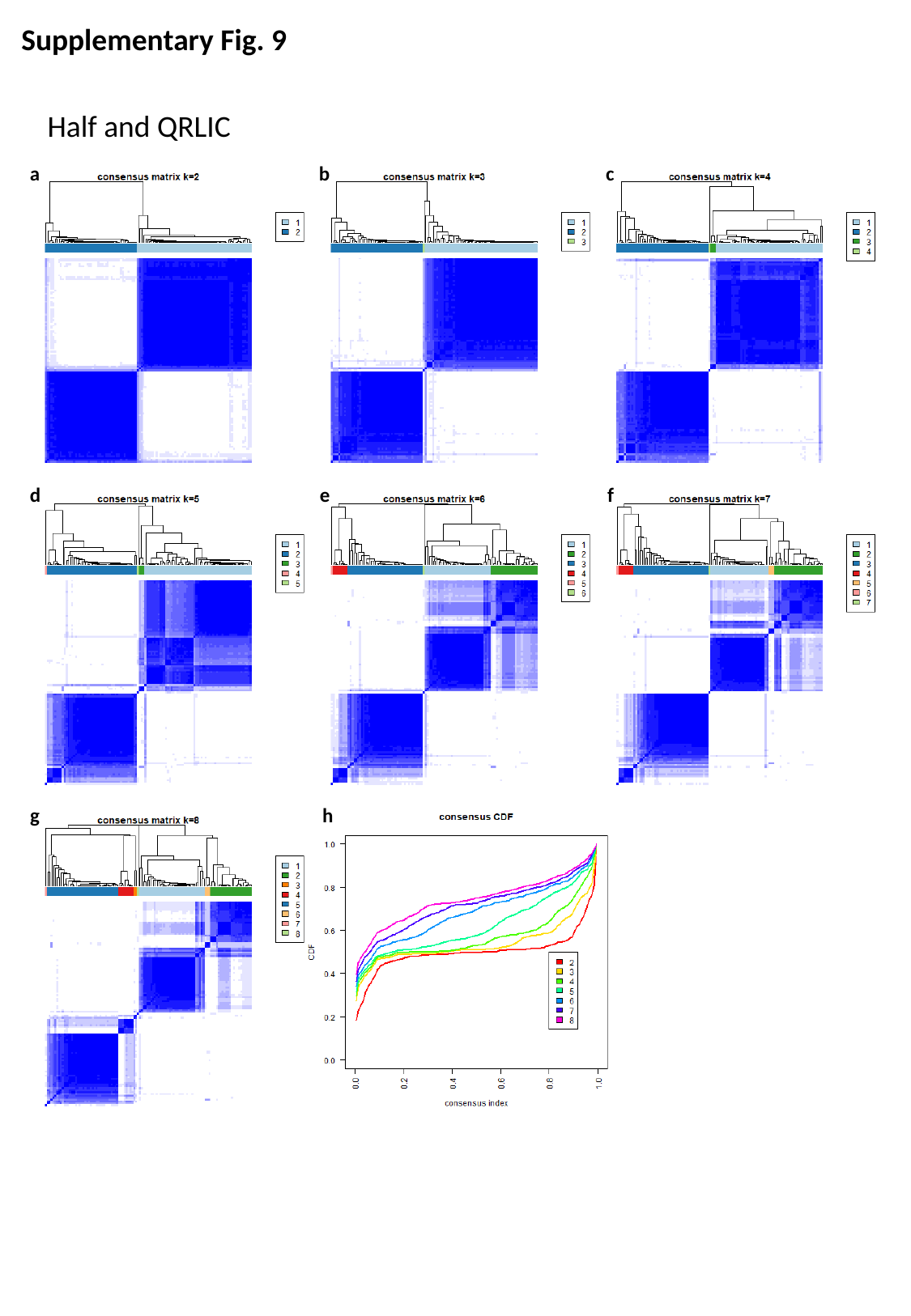

Supplementary Fig. 9
Half and QRLIC
a
b
c
d
e
f
g
h

Supplement: Supplementary file 19 — Figure S9 [file CAM4-12-7616-s007.pptx]
