# Supplementary material for: A large‐scale targeted proteomics of plasma extracellular vesicles shows utility for prognosis prediction subtyping in colorectal cancer
Source: Cancer Med. 2022 Nov 16;12(6):7616–26. doi: 10.1002/cam4.5442 (PMC10067095; doi:10.1002/cam4.5442)
Supplement: Supplementary file 20 — Figure S10 [file CAM4-12-7616-s014.pptx]

## Slide 1
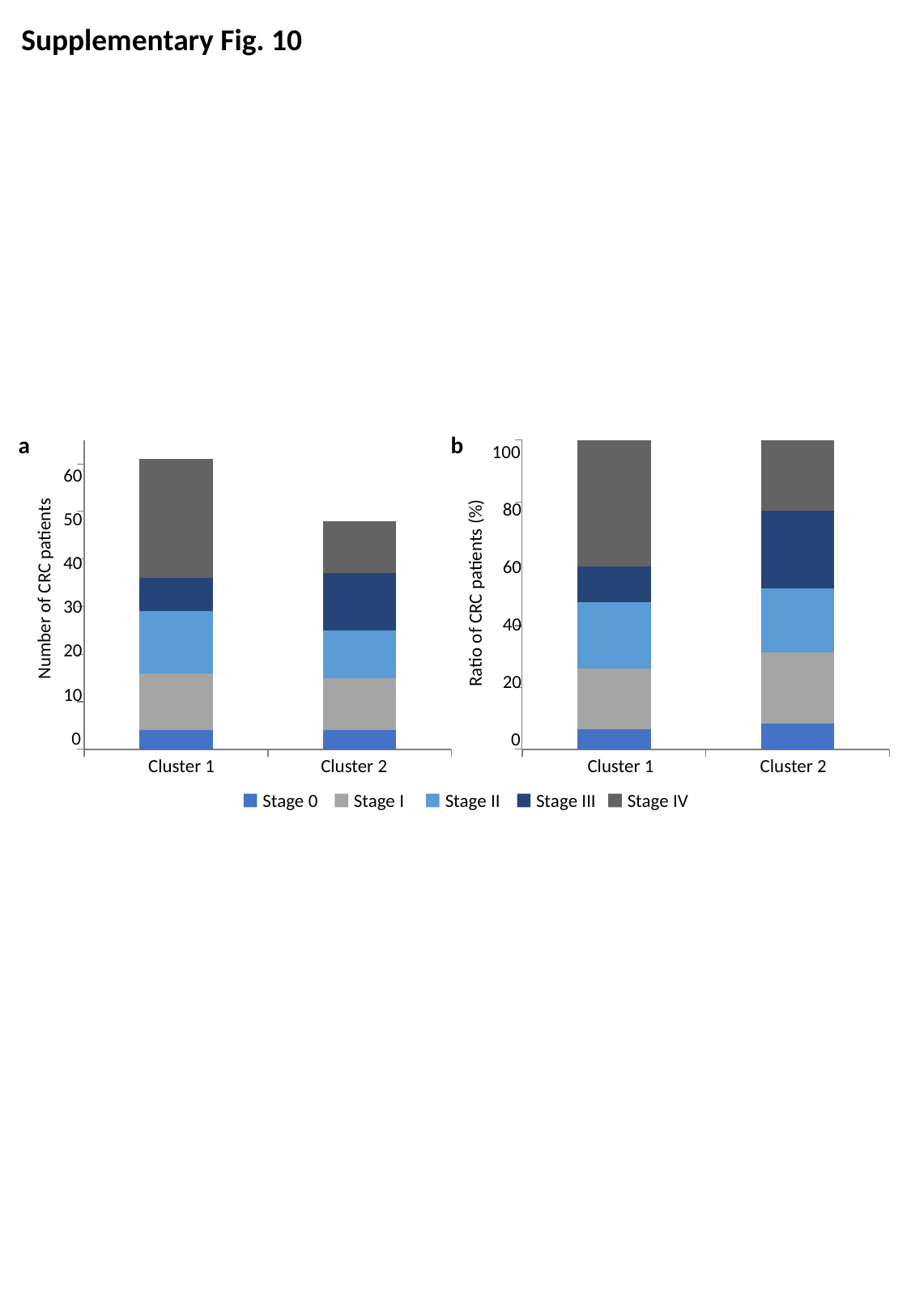

Supplementary Fig. 10
a
b
### Chart
| Category | Stage 0 | Stage I | Stage II | Stage III | Stage IV |
|---|---|---|---|---|---|
| cluster 1 | 4.0 | 12.0 | 13.0 | 7.0 | 25.0 |
| cluster 2 | 4.0 | 11.0 | 10.0 | 12.0 | 11.0 |60
50
40
30
20
10
0
Cluster 2
Cluster 1
### Chart
| Category | Stage 0 | Stage I | Stage II | Stage III | Stage IV |
|---|---|---|---|---|---|
| cluster 1 | 4.0 | 12.0 | 13.0 | 7.0 | 25.0 |
| cluster 2 | 4.0 | 11.0 | 10.0 | 12.0 | 11.0 |100
80
60
40
20
0
Cluster 1
Cluster 2
Number of CRC patients
Ratio of CRC patients (%)
Stage 0
Stage I
Stage II
Stage III
Stage IV
