# Supplementary material for: A large‐scale targeted proteomics of plasma extracellular vesicles shows utility for prognosis prediction subtyping in colorectal cancer
Source: Cancer Med. 2022 Nov 16;12(6):7616–26. doi: 10.1002/cam4.5442 (PMC10067095; doi:10.1002/cam4.5442)
Supplement: Supplementary file 21 — Figure S11 [file CAM4-12-7616-s017.pptx]

## Slide 1
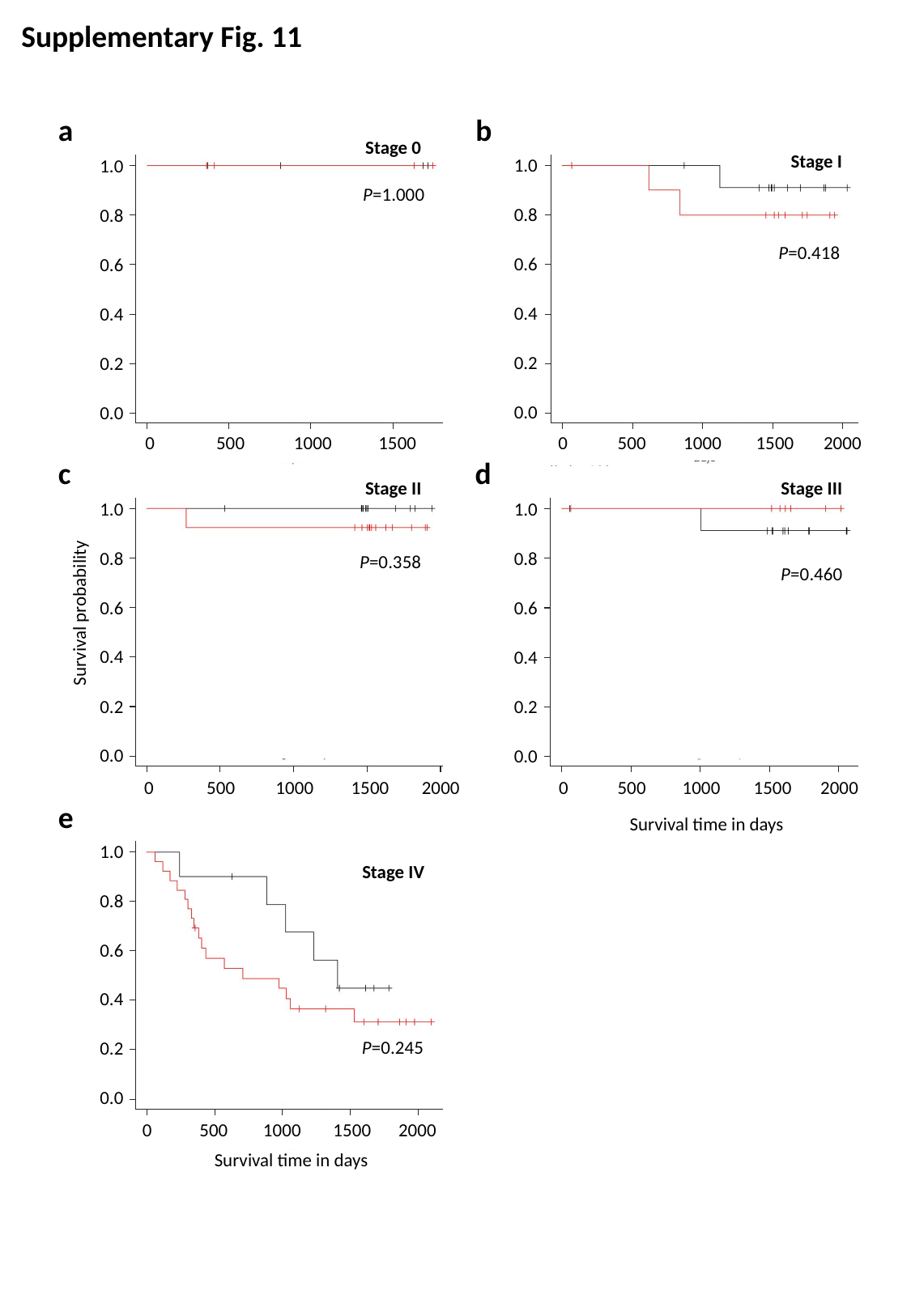

Supplementary Fig. 11
a
b
Stage 0
Stage I
1.0
1.0
P=1.000
0.8
0.8
P=0.418
0.6
0.6
0.4
0.4
0.2
0.2
0.0
0.0
0
500
1000
1500
0
500
1000
1500
2000
c
d
Stage II
Stage III
1.0
1.0
0.8
0.8
P=0.358
P=0.460
0.6
0.6
Survival probability
0.4
0.4
0.2
0.2
0.0
0.0
0
500
1000
1500
2000
0
500
1000
1500
2000
e
Survival time in days
1.0
Stage IV
0.8
0.6
0.4
P=0.245
0.2
0.0
0
500
1000
1500
2000
Survival time in days
