# Supplementary material for: A large‐scale targeted proteomics of plasma extracellular vesicles shows utility for prognosis prediction subtyping in colorectal cancer
Source: Cancer Med. 2022 Nov 16;12(6):7616–26. doi: 10.1002/cam4.5442 (PMC10067095; doi:10.1002/cam4.5442)
Supplement: Supplementary file 22 — Figure S11 [file CAM4-12-7616-s018.docx]

**Supporting Information**

**SI Results and discussions**

***Experimental design***

CRC patient samples were collected from two independent cohorts at separate hospitals and at different times to improve the reliability of the analyses for understanding the actual molecular-level phenomenon (Supplementary **Fig. 1**). Both early-stage (Stage I) and advanced-stage (Stage IV) patients were selected in cohort 1 according to the original criteria based on the TNM classification with pathological and CT/MR imaging findings. Healthy controls (HC1 and HC2) were selected to match the sex and age of the patients (**Supplementary Data 1**). The patients in cohort 2 were selected at another hospital over a certain period of time irrespective of cancer stage. After the acquisition of proteome data, 40 patients in cohort 2 were excluded because they did not meet the eligibility criteria (see **SI Materials and Methods**).

Large-scale SRM-based targeted proteomics analyses performed by the absolute quantification of 1,344 peptides representing 457 proteins, and 139 peptides representing 99 proteins, were examined in studies of cohorts 1 and 2, respectively. A list of biomarker candidates was derived from the cohort 1 study, which showed significant changes in the quantified amount of plasma EVs between CRC patients and healthy controls and between early- and advanced-stage patients. The Mann-Whitney-Wilcoxon (MWW) test was used for statistical analysis, and the random forest (RF) machine learning algorithm ^1^ was also applied to reinforce the statistical analyses.

The obtained list was applied in the cohort 2 study, which aimed to investigate the association between individual markers and the disease status of the patients. The patients were classified according to a set of quantified values for 99 selected proteins. Consensus clustering (CC) ^2^ was used to group individuals based on similarities of protein levels. The Kaplan-Meier survival curve and multivariate Cox proportional hazard model were applied to address potential molecular factors contributing to the survival of each patient.

**Supplementary Fig. 2** shows the workflow of the proteome analysis of plasma EVs performed in the present study. EVs were isolated from plasma by ultracentrifugation with a sucrose cushion (UC in **Supplementary Fig. 3a**), which showed the most sensitive detection of EVs markers listed in **Supplementary Fig. 3c**. The amounts of all five markers were relatively higher than those of the other methods (**Supplementary Fig. 3d**), whereas the total protein amount showed the lowest value (**Supplementary Fig. 3b**). Absolute quantification was carried out by using SIL peptides as internal standards, which were prepared via an improved MS-QBiC method ^3^ based on the original one^4, 5^ . The method is based on the multiplexed cell-free production of SIL peptides, and thus, over 1,000 SIL peptides were prepared in a low-cost and time-saving manner (**Supplementary Fig. 4**). Note that SRM method development for the optimized monitoring of each peptide transition can be performed by using the synthesized SIL peptides. EVs proteins were digested with SIL peptides, fractionated to improve the sensitivity of the analysis by reducing sample complexity^6^, and subjected to liquid chromatography (LC)-SRM analysis.

**Impact of large-scale targeted proteomics on biomarker discovery research**

MS-based proteomics has become a powerful technology for biomarker discovery research. A current standard approach is based on the integration of non-targeted and targeted analyses. Non-targeted analysis can quantify the near-comprehensive proteome with limited precision, which is suitable for maximizing the coverage of potential biomarker candidates. The identified candidates were further validated with targeted proteome analysis based on the application of SRM ^7, 8, 9^.

The development of large-scale SRM assays with a large set of SIL peptides can provide various benefits to biomarker discovery studies. Greater numbers of proteins can be quantified with sufficient quality, which means that the step corresponding to validation in general integrated approaches can be performed for many biomarker candidates. Furthermore, increasing the number of SIL peptides per target protein can lead to an improved success rate for target quantification. In the present study, 1,344 SIL peptides representing 457 proteins were designed, but only 563 peptides representing 282 proteins were detected in the mixtures of plasma samples, and 230 peptides representing 162 proteins were successfully quantified in the cohort 1 study (Supplementary Data 2, 3), indicating that a large proportion of the designed peptides were unsuitable and discarded. The MS signal intensity of the peptides highly depends on their peptide sequence; therefore, a sufficient variety of peptides must be considered.

Various methodologies for providing SIL peptides at a lower cost and in a more high-throughput manner have been proposed to date^5^, including the use of multiplexed chemical peptide synthesis ^10^, cellular artificial protein expression composed of a concatenation of target peptides (QconCAT) ^11^, and cell-free expression systems ^12, 13^. In line with these advancements, we previously developed the MS-QBiC method for multiplexed synthesis of SIL peptides and revealed the expression dynamics of circadian clock proteins using 120 SIL peptides representing 20 proteins ^4^.

The present study applied this method to a more large-scale clinical study. To the best of our knowledge, this is one of the largest-scale clinical studies, which investigated more than 200 clinical plasma samples with more than 1,000 synthesized SIL peptides, proving that our strategy is advantageous for increasing the coverage of proteins with SRM-based analysis. Apart from clinical studies, same-level or even larger-scale SRM studies have been addressed with library-based approaches. cDNA libraries encoding human proteins or QconCAT libraries encoding human and yeast proteotypic peptides have been used ^12, 13, 14^. Once a library is constructed, library-based approaches can provide solid benefits to the study of the target organisms. However, accessing these libraries can be a difficult task, and working with them can also be problematic, e.g., selecting the desired set of proteins to be analyzed or keeping them in individual laboratories.

In contrast, the method presented here can be performed from scratch, starting only with the sequence information of the peptides to be analyzed. After designing oligo DNAs for peptide production (Supplementary Data 8), PCR, cell-free peptide synthesis, and peptide purification steps can be performed seamlessly, and SIL peptides can be obtained in at least one day (Supplementary Fig. 3), providing an easy-to-access technique in SRM-based clinical studies in the future. Currently, a large part of the cost of acquiring SIL peptides in our method depends on the price of oligo DNAs, with approximately ten to twenty dollars needed for one peptide. However, oligo DNA synthesis technologies are currently in a period of remarkable development ^15, 16^, and it is conceivable that this method could be improved by combining oligo DNA synthesis with these technologies in terms of the cost and diversity of SIL peptides. Additionally, methods using small-scale batch reactions, including PCR and cell-free reactions, are compatible with laboratory automation and thus have the possibility to become part of the standard methodology for SRM-based analysis.

**SI Materials and Methods**

***Sample collection***

Plasma samples were obtained from CRC patients who had no history of other malignant diseases and from healthy controls who were not taking any medications and with no past history of malignant diseases. Plasma samples were collected from cohort 1 between April 2011 and December 2012 (CRC patients) and October 2017 and July 2018 (healthy controls) at The Cancer Institute Ariake Hospital of Japanese Foundation of Cancer Research. This cohort included 26 CRC patients with stage I disease, 33 CRC patients with stage IV disease, and 59 age- and sex-matched healthy controls (**Supplementary Data 1**). CRC patients were selected according to the following criteria: (a) no anticancer treatment before blood sample collection, (b) stage I disease with no evidence of pathological lymphovascular invasion and three-year disease-free survival after complete surgical resection, and (c) stage IV disease with pathologically proven lung metastasis or pathologically proven liver metastasis or disease confirmed by CT or MR images. Plasma samples were collected from cohort 2, comprising 150 CRC patients of all disease stages, between September 2013 and December 2015 and were provided by Clinical Bio-Resource Center, Kyoto University Hospital. Only information on the registered disease, colorectal cancer diagnosis and anticancer therapies were provided when choosing the samples. All clinical information, including sex, age, TNM stage, etc., was obtained after termination of the sample transfer. After acquiring the proteomic data, CRC patients were selected according to the following criteria: (a) no anticancer treatment before blood sample collection and (b) pathologically proven colorectal cancer. The collected plasma samples were stored at -80°C until use.

***Extracellular vesicles isolated from plasma***

EVs were isolated from 100 μL of plasma by the cushioned ultracentrifugation method. The plasma sample was diluted fivefold with PBS and centrifuged at 300 *g* for 10 min to remove debris, and then the supernatant was passed through a 0.22 μm spin filter (Agilent Technologies, USA). An aliquot of the resultant sample was overlaid onto 350 μL of PBS and 250 μL of 30% sucrose/deuterium oxide (For Nuclear Magnetic Resonance, FUJIFILM Wako, Japan) solution, followed by ultracentrifugation at 100,000 *g* for 90 min at 4°C using a P56ST swing rotor (HITACHI, Japan). After aspirating 200 μL of the EVs-enriched fraction from the bottom of the centrifugation tube using a gel-loading tip, the remaining solution was discarded. A pelleted fraction was further recovered by resuspending the pellet with 1,000 μL of PBS, which was mixed with 200 μL of the EVs-enriched fraction. The resultant sample was ultracentrifuged at 100,000 g for 70 min twice, and finally, the pellet was resuspended in 50 μL phase-transfer surfactant (PTS) buffer composed of 50 mM NH_4_HCO_3_, 12 mM sodium deoxycholate, and 12 mM sodium *N*-lauroyl sarcosinate^17^. Other isolation methods using commercially available kits (**Supplementary Fig. 3**) were performed according to the manufacturer’s instructions.

***Preparation of peptide samples for MS analysis***

Proteins in the isolated EVs fractions were reduced with 10 mM TCEP for 30 min at 37°C and then alkylated with 20 mM iodoacetamide for 30 min at 37°C, which were subsequently digested with 2 mAU LysC (Wako-Chemical, Tokyo, Japan) and 1 μg of trypsin (Proteomics grade, Roche, Mannheim, Germany) at 37°C overnight. The digested solutions were acidified with 1% TFA and centrifuged at 20,000 *g* for 10 min to precipitate the detergents. Supernatants containing the digested peptides were separated into seven fractions for non-targeted analysis or into two fractions for SRM analysis using pipette-tip columns with C18 and SCX resins, as previously described^18^. Cell-free synthesized SIL peptides were spiked into the samples just before the digestion procedure.

***Preparation of SIL peptides***

Plasmids with sequences (5’- ATGGACTACAAGGACGACGACGACAAGCTGCTGCTGCTGAAGCTGGTTACTXXXCTGACTAAG -3’) that encode a FLAG-tag (MDYKDDDDK), spacer peptide (LLLLK), and quantification tags (LVTXLTK), downstream of a T7 promoter sequence and the ribosome binding site (Shine-Dalgarno sequence), were used for amplification of the DNA templates for SIL peptide synthesis using an improved MS-QBiC method ^3^. X represents one of the 12 amino acids (Ala, Asp, Glu, Phe, Gly, His, Leu, Pro, Ser, Thr, Val, or Tyr), and XXX represents the codons corresponding to the 12 amino acids. Using these plasmids as templates, DNA templates were amplified by one- or two-step PCR with a T7 promoter primer (5'-GGGCCTAATACGACTCACTATAG-3') as a forward primer and the appropriate reverse primers, as listed in **Supplementary Data 8**. By utilizing the variation in quantification tags, each of the 12 SIL peptides was synthesized in a one-pot cell-free reaction, where 12 template DNAs were added to each of the PURE system reaction mixtures. The PURE reaction mixtures contained ^13^C_6_ ^15^N_4_ L-arginine (Thermo Scientific) and ^13^C_6_ ^15^N_2_ L-lysine (Silantes) as substitutes for nonlabelled L-arginine and L-lysine, respectively, for stable isotope labeling. Procedures for cell-free synthesis and subsequent purification have been described in a previous report ^4^.

***MS analysis***

Non-targeted LC-MS/MS analysis was performed with an Orbitrap mass spectrometer (scan range of 350-1,800 *m/z*, 70,000 resolution after the accumulation of ions to a 3×10^6^ target value; Q-Exactive mass spectrometer, Thermo Scientific, Bremen, Germany), while targeted SRM analysis was performed with a triple quadruple mass spectrometer (scan width of 0.002 *m/z*, Q1 and Q3 resolutions of 0.7 FWHM, cycle time of 2.5 s, gas pressure of 1.8 m Torr; TSQ-Vantage mass spectrometer, Thermo Scientific, Bremen, Germany). Each mass spectrometer was connected to an UltiMate 3000 Nano-flow high-performance LC system (Thermo Fisher Dionex, Sunnyvale, CA) with an analytical column that was packed with C18 material (ReproSil-Pur C18-AQ, 1.9-μm resin, Dr. Maisch, Ammerbuch-Entringen, Germany) into a needle (length 300 mm for non-targeted LC-MS/MS or 100 mm for targeted SRM analysis, inner diameter 75 μm) and HTC-PAL autosampler (CTC Analytics, Zwingen, Switzerland) with a trap column (30 μm x 10 cm, nanoViper; Thermo Scientific, Bremen, Germany). Samples were separated at a flow rate of 280 nL/min using two mobile phases A (0.1% formic acid and 2% acetonitrile) and B (0.1% formic acid and 90% acetonitrile) with a gradient of 5-35% B for 105 min (non-targeted LC-MS/MS) or 30 min (targeted SRM analysis). Non-targeted LC-MS/MS data were analyzed with MaxQuant version 1.5 ^19^. The precursor mass tolerance was 7 ppm, and the fragment ion mass tolerance was 0.01 Da. Peptides and proteins were identified with a false discovery rate of < 1%. Targeted SRM data were analyzed with Skyline ^20^. SRM signal peaks corresponding to each target peptide were assigned by comparison with the SIL internal standard for each counterpart. The absolute quantitative values of the target peptide were obtained as ratios of the endogenous target peptide to the SIL internal standard using the highest signal transition. All transitions estimated from the designed peptide sequence were tested by pooled EVs fractions, and the top five high-intensity transitions for each peptide were selected for quantification (**Supplementary Data 2**).

***Statistical analysis***

Statistical analyses were performed with R (http://cran.r-project.org/). The Mann-Whitney-Wilcoxon (MWW) test was performed to calculate p-values for each biomarker candidate in three comparative groups in cohort 1 and two comparative groups obtained by consensus clustering in cohort 2 using R’s Wilcox test function. P-values were adjusted using R’s p.adjust function. A survival analysis and Cox proportional analysis were performed using the survival package in EZR (Easy R), an R commander for statistical graphical user interfaces ^21^. Overall survival (OS) was defined the time from the date of diagnosis by computed tomography or colonoscopy to the date of death. OS was analyzed using the Kaplan-Meier survival curve method, and differences in OS were examined using the log-rank test in cohort 2, in which patients were stratified based on clusters by consensus clustering or median protein levels (above median = high, below median = low). A Cox proportional hazards model was used to estimate multivariate hazard ratios for OS.

***Random forest***

Random forest (RF) was performed using the RandomForest package in R to calculate distances between samples in cohort 1 ^1^ . RF predictor is an ensemble of individual decision tree predictors. The method can use for unlabeled data as unsupervised learning. The idea is to construct an RF predictor that distinguishes the observed data from suitably generated synthetic data. RF training run for these two data and proportion of separated observed data in the same terminal node are calculated as the similarity of each sample. The similarities between observed samples are aligned as a matrix with follow features: symmetric, positive, and each entry lies in the interval from 0 to 1. Using this matrix, the RF dissimilarity matrix is calculated by 1 – similarity and used like as distances between each other. Three parameters, including the number of trees, number of variables randomly sampled, and minimum size of the terminal node, were set to 2,000, 100, and 1, respectively. Calculated dissimilarities among samples were used as inputs for generating a multi-dimensional scaling plot.

***Consensus clustering***

Consensus clustering (CC) was performed using ConsensusClusterPlus, (ver. 1.50.0) in R^2^ . The parameters for CC were as follows: the ratio of item resampling was 0.9; the ratio of feature resampling was 0.9; the number of subsamples was 200; and the clustering algorithm was hierarchical clustering and Pearson’s distance.

***Imputation of missing values***

Missing value imputation was performed using imputeLCMD (ver. 2.0) in R. Four methods were applied as follows: Features were removed if there was at least one missing value (Removing). All missing values were replaced with zero (Zero). Missing values were replaced with half the value of the minimum protein level (Half). A combination of Half and QRLIC (Half and QRLIC). QRILC (quantile regression imputation of left-censored data) imputation was specifically developed for left-censored data^22^. Log-transformation for measurements was performed before QRLIC to improve the imputation accuracy.

***Correlation analysis***

Correlations between protein levels in cohort 2 were analysed using the Hmisc package in R to the calculate correlation coefficients and the p-values with the pairwise complete observations option to allow for missing data. Correlations using fewer than 10 data points were subsequently removed. False discovery rates were estimated using the fdrtool package in R. To construct the correlation network, correlations of the 12 upregulated proteins in cluster 1 with FDR < 10^-4^ were extracted. The resulting proteins and correlations, which were used as nodes and edges of the correlation network, respectively, were visualized with Cytoscape (https://cytoscape.org/)

**SUPPLEMENTARY DATE**

**Supplementary Data 1. Features of the individuals in cohort 1 and 2.**

**Supplementary Data 2. List of the target proteins and peptides analysed and their SRM transitions.**

**Supplementary Data 3. Proteomic data from the cohort 1 and 2 studies.**

**Supplementary Data 4. Proportion of ambiguous clustering (PAC) values.**

**Supplementary Data 5. Comparison of the 2 clusters identified by consensus clustering with a focus on curative resection in Stage IV patients in cohort 2.**

**Supplementary Data 6. Cox proportional hazards regression analysis of the 110 patients in cohort 2.**

**Supplementary Data 7. Cox proportional hazards regression analysis of the stage IV patients in cohort 2.**

**Supplementary Data 8. List of the oligo DNA primers used for cDNA amplification of the templates coding the target peptides.**

**Supplementary Data 9. List of gene ontology (GO) terms that are related to the upregulated proteins in cluster 1 obtained by consensus clustering.**

**Supplementary Data 10. List of significant protein pairs including twelve upregulated proteins.** Pairs with a false discovery rate < 1.0 × 10^-4^ are listed.

**Supplementary Figure 1. Experimental design.** Diagram showing the experimental design of this study. Cohort 1 samples consisted of 26 stage I CRC patients and 26 sex- and age-matched healthy controls (HC1) and 33 stage IV CRC patients and 33 sex- and age-matched healthy controls (HC2). A large-scale targeted proteomics study of plasma EVs was performed with 1,344 peptides to quantify 457 CRC biomarker candidate proteins. The Mann-Whitney-Wilcoxon (MWW) test and random forest (RF) analyses were performed to select biomarker candidates, which were further analysed in cohort 2. Cohort 2 samples consisted of 150 CRC patients irrespective of TNM staging. Target proteomics analyses were performed with 139 peptides to quantify 99 biomarker candidate proteins. After the acquisition of proteome data, 40 patients were excluded (shown in grey and enclosed in the dashed line) because they did not meet the eligibility criteria (see **Methods**). The EV protein levels of 110 patients were analysed by consensus clustering (CC). Kaplan-Meier and Cox proportional hazard model analyses were performed to characterize the identified clusters with CC.

**Supplementary Figure 2. Workflow of the targeted proteomics analysis of plasma EV proteins.** EVs were prepared for each individual. SIL peptides were prepared with a cell-free protein synthesis system. They were combined and enzymatically digested to obtain tryptic peptides. The peptides were pre-fractionated, and each fraction was analysed with LC-SRM. Comparison of light (native peptides) and heavy (SIL peptides) peptide peaks enables absolute quantification.

**Supplementary Figure 3. Optimization of EV isolation.** Several methods for EV isolation were evaluated. The method using ultracentrifugation with a sucrose cushion (UC) showed the highest level of EV marker protein, while the level of total protein was the lowest, suggesting the purity of EVs obtained by this method. (a) Table of the EV isolation methods compared in this study. (b) Total amount of protein contained in isolated EVs. EVs were isolated from 100 μL of plasma by each method. (c) Table of EV marker proteins and their target peptides quantified in this study. (d) Relative amount of EV marker proteins according to the quantified values of each peptide.

**Supplementary Figure 4. Schematic description of the MS-QBiC workflow.** The SIL peptide (MS-QBiC peptide) is synthesized from PCR-amplified cDNA encoding the MS-QBiC peptide sequence. SIL amino acids (Arg and Lys) are added to a reconstituted cell-free protein synthesis system (PURE), and SIL MS-QBiC peptides are synthesized. The MS-QBiC peptide consists of a purification tag, quantification tag, and target peptide, which are tandemly arranged. The quantification tag was used for the absolute quantification of the synthesized MS-QBiC peptide by using chemically synthesized identical peptides. The obtained quantified value was applied for target peptide quantification.

**Supplementary Figure 5. Random forest analysis of each individual EV protein profile in cohort 1.** Multidimensional scaling plots according to the similarities of each individual’s EV protein profiles when analysed with RF. The plots were derived with two datasets: (a) patient data (Stages I and IV) and (b) all acquired data (Stages I, IV and HC1, 2).

**Supplementary Figure 6. Detailed results of consensus clustering of cohort 2 patients.** Consensus matrix plots are shown when the maximum number of clusters (*k*) was (a) *k*=3, (b) *k*=4, (c) *k*=5, (d) *k*=6, (e) *k*=7, and (f) *k*=8. (g) Item tracking plot showing traces of clusters when *k* was increased from two to eight.

**Supplementary Figure 7. Consensus clustering with imputation of missing values via “Removing”.** The imputation method “Removing” removes features if there is at least one missing value. Consensus matrix plots are shown when the maximum number of clusters (*k*) was (a) *k*=2, (b) *k*=3, (c) *k*=4, (d) *k*=5, (e) *k*=6, (f) *k*=7 and (g) *k*=8. (h) Empirical cumulative distribution function plots when *k* was increased from two to eight.

**Supplementary Figure 8. Consensus clustering with imputation of missing values via “Zero”.** The imputation method “Zero” replaces all missing values with zero. Consensus matrix plots are shown when the maximum number of clusters (*k*) was (a) *k*=2, (b) *k*=3, (c) *k*=4, (d) *k*=5, (e) *k*=6, (f) *k*=7 and (g) *k*=8. (h) Empirical cumulative distribution function plots when *k* was increased from two to eight.

**Supplementary Figure 9. Consensus clustering with imputation of missing values via “Half and QRLIC”.** The imputation method “Half” replaces missing values with half values of the minimum protein level. The method “Half and QRLIC” is the combination of “Half” and Quantile Regression imputation of Left-Censored data^36^. Consensus matrix plots are shown when the maximum number of clusters (*k*) was (a) *k*=2, (b) *k*=3, (c) *k*=4, (d) *k*=5, (e) *k*=6, (f) *k*=7 and (g) *k*=8. (h) Empirical cumulative distribution function plots when *k* was increased from two to eight.

**Supplementary Figure 10. Distribution of TNM staging in the two clusters identified by consensus clustering.** (a) The number and (b) ratio of patients with each TNM stage in the two clusters are shown.

**Supplementary Figure 11. Overall survival curves of patients in each TNM stage in the two clusters identified by consensus clustering.** (a-e) Kaplan-Meier OS curves of each TNM stage patient in two clusters: (a) Stage 0, (b) Stage I, (c) Stage II, (d) Stage III and (e) Stage IV. The red and black curves indicate cluster 1 and 2, respectively.

**References**

1 Breiman L. Random Forest. *Machine Learning*. 2001; 45: 5-32.

2 Wilkerson MD, Hayes DN. ConsensusClusterPlus: a class discovery tool with confidence assessments and item tracking. *Bioinformatics*. 2010; 26: 1572-1573.

3 Masuda K, Kasahara K, Narumi R, Shimojo M, Shimizu Y. Versatile and multiplexed mass spectrometry-based absolute quantification with cell-free-synthesized internal standard peptides. *J Proteomics*. 2022; 251: 104393.

4 Narumi R, Shimizu Y, Ukai-Tadenuma M, et al. Mass spectrometry-based absolute quantification reveals rhythmic variation of mouse circadian clock proteins. *Proc Natl Acad Sci U S A*. 2016; 113: E3461-3467.

5 Narumi R, Masuda K, Tomonaga T, Adachi J, Ueda HR, Shimizu Y. Cell-free synthesis of stable isotope-labeled internal standards for targeted quantitative proteomics. *Synth Syst Biotechnol*. 2018; 3: 97-104.

6 Burgess MW, Keshishian H, Mani DR, Gillette MA, Carr SA. Simplified and efficient quantification of low-abundance proteins at very high multiplex via targeted mass spectrometry. *Mol Cell Proteomics*. 2014; 13: 1137-1149.

7 Vasaikar S, Huang C, Wang X, et al. Proteogenomic Analysis of Human Colon Cancer Reveals New Therapeutic Opportunities. *Cell*. 2019; 177: 1035-1049 e1019.

8 Picotti P, Bodenmiller B, Aebersold R. Proteomics meets the scientific method. *Nat Methods*. 2013; 10: 24-27.

9 Kume H, Muraoka S, Kuga T, et al. Discovery of colorectal cancer biomarker candidates by membrane proteomic analysis and subsequent verification using selected reaction monitoring (SRM) and tissue microarray (TMA) analysis. *Mol Cell Proteomics*. 2014; 13: 1471-1484.

10 Picotti P, Rinner O, Stallmach R, et al. High-throughput generation of selected reaction-monitoring assays for proteins and proteomes. *Nat Methods*. 2010; 7: 43-46.

11 Pratt JM, Simpson DM, Doherty MK, Rivers J, Gaskell SJ, Beynon RJ. Multiplexed absolute quantification for proteomics using concatenated signature peptides encoded by QconCAT genes. *Nat Protoc*. 2006; 1: 1029-1043.

12 Matsumoto M, Matsuzaki F, Oshikawa K, et al. A large-scale targeted proteomics assay resource based on an in vitro human proteome. *Nat Methods*. 2017; 14: 251-258.

13 Takemori N, Takemori A, Tanaka Y, et al. MEERCAT: Multiplexed Efficient Cell Free Expression of Recombinant QconCATs For Large Scale Absolute Proteome Quantification. *Mol Cell Proteomics*. 2017; 16: 2169-2183.

14 Lawless C, Holman SW, Brownridge P, et al. Direct and Absolute Quantification of over 1800 Yeast Proteins via Selected Reaction Monitoring. *Mol Cell Proteomics*. 2016; 15: 1309-1322.

15 Hughes RA, Ellington AD. Synthetic DNA Synthesis and Assembly: Putting the Synthetic in Synthetic Biology. *Cold Spring Harb Perspect Biol*. 2017; 9.

16 Perkel JM. The race for enzymatic DNA synthesis heats up. *Nature*. 2019; 566: 565.

17 Masuda T, Tomita M, Ishihama Y. Phase transfer surfactant-aided trypsin digestion for membrane proteome analysis. *J Proteome Res*. 2008; 7: 731-740.

18 Adachi J, Hashiguchi K, Nagano M, et al. Improved Proteome and Phosphoproteome Analysis on a Cation Exchanger by a Combined Acid and Salt Gradient. *Anal Chem*. 2016; 88: 7899-7903.

19 Tyanova S, Temu T, Cox J. The MaxQuant computational platform for mass spectrometry-based shotgun proteomics. *Nat Protoc*. 2016; 11: 2301-2319.

20 MacLean B, Tomazela DM, Shulman N, et al. Skyline: an open source document editor for creating and analyzing targeted proteomics experiments. *Bioinformatics*. 2010; 26: 966-968.

21 Kanda Y. Investigation of the freely available easy-to-use software 'EZR' for medical statistics. *Bone Marrow Transplant*. 2013; 48: 452-458.

22 Wei R, Wang J, Su M, et al. Missing Value Imputation Approach for Mass Spectrometry-based Metabolomics Data. *Sci Rep*. 2018; 8: 663.
